# Remdesivir alleviates skin fibrosis by suppressing TGF-β1 signaling pathway

**DOI:** 10.1371/journal.pone.0305927

**Published:** 2024-07-18

**Authors:** Jianwei Zhang, Xiujun Zhang, Xiaowei Guo, Wenqi Li, Tiantian Zhang, Dan Chai, Yuming Liu, Li Chen, Xiaoyu Ai, Tianyuan Zhou, Wenguo Wei, Xiaoting Gu, Xiaohe Li, Honggang Zhou

**Affiliations:** 1 State Key Laboratory of Medicinal Chemical Biology, College of Pharmacy, College of Life Sciences, Nankai University, Tianjin, China; 2 Tianjin Key Laboratory of Molecular Drug Research, Tianjin International Joint Academy of Biomedicine, Tianjin, China; 3 Department of Dermatology, Tianjin Academy of Traditional Chinese Medicine Affiliated Hospital, Tianjin, China; 4 Department of Dermatology, Tianjin First Central Hospital, School of Medicine, Nankai University, Tianjin, China; Pennsylvania State University Hershey Medical Center, UNITED STATES OF AMERICA

## Abstract

Fibrotic skin diseases, such as keloids, are pathological results of aberrant tissue healing and are characterized by overgrowth of dermal fibroblasts. Remdesivir (RD), an antiviral drug, has been reported to have pharmacological activities in a wide range of fibrotic diseases. However, whether RD function on skin fibrosis remains unclear. Therefore, in our study, we explored the potential effect and mechanisms of RD on skin fibrosis both *in vivo* and *in vitro*. As expected, the results demonstrated that RD alleviated BLM‐induced skin fibrosis and attenuates the gross weight of keloid tissues *in vivo*. Further studies suggested that RD suppressed fibroblast activation and autophagy both *in vivo* and *in vitro*. In addition, mechanistic research showed that RD attenuated fibroblasts activation by the TGF‐β1/Smad signaling pathway and inhibited fibroblasts autophagy by the PI3K/Akt/mTOR signaling pathway. In summary, our results demonstrate therapeutic potential of RD for skin fibrosis in the future.

## 1. Introduction

Skin fibrosis is a pathological result of aberrant tissue healing following deep dermal skin injury, accompanied by marked expansion of the dermis with obliteration [1], and can occur in various pathological processes, including keloids, hypertrophic scars, and immunological diseases such as scleroderma. For instance, excessive extracellular matrix (ECM), such as collagen, is deposited in the dermis and subcutaneous tissues due to human skin injuries, burns, or surgery, leading to keloids, a skin fibrotic illness. Keloids are similar with benign tumor that appears as abnormal fibrous proliferations on the surface of the skin [2], which usually originate from excessive repair of skin wounds and skin inflammation. Patients often experience discomfort, such as pruritus, intermittent painness and a feeling of tightness [3]. Heavy sufferers may have some functional impairment, which affects their quality of life and even has a negative effect on mental health in severe cases.

At present, treatments for keloids are still an unsolved problem worldwide [4]. The readily available drugs and methods for treating pathological scars are limited mainly due to incomplete understanding of the mechanism of scar formation. Recent research has demonstrated that the basic basis of keloid formation is a combination of extracellular matrix deposition, excessive fibroblast proliferation, and inflammatory cell infiltration during tissue repair [5]. During keloid formation, fibroblasts, as the primary effector cells, play a key role in leading to a persistent inflammatory response and excessive ECM deposition [6,7]. Meanwhile, this process is driven by many growth factors, including transforming growth factor-β (TGF-β), which is generally considered the most critical regulatory factor [8,9]. The TGF-β signaling pathway is the most basic signaling pathway in maintaining life activities, and it is also one of the most critical signaling pathways in fibrosis [10]. Moreover, accumulating evidence suggests that autophagy is closely associated with the pathogenesis and treatment of keloids. Some research has demonstrated that autophagy is disturbed in keloid fibroblasts, leading to significantly reduced autophagic flux in keloid fibroblasts. These results suggest that the induction of autophagy may have therapeutic efficacy in keloids [11,12].

Remdesivir (RD) is a nucleoside analog drug that inhibits viral RNA-dependent RNA polymerase (RdRP) through triphosphate metabolites, which could inhibit the transcription of viral RNA and prevent viral replication. In 2015, the FDA approved RD for the treatment of Ebola virus [13]. Although RD was developed as an antiviral drug and most of the current research has focused on its antiviral activity, some studies have focused on the efficacy and pharmacological activity of RD in other diseases. Our previous studies have shown that RD could attenuate bleomycin-induced pulmonary fibrosis in mice by suppressing TGF-β1-induced lung fibroblast activation [14]. The antifibrotic effects of RD have also been reported in renal fibrosis and liver fibrosis [15,16]. These studies indicate that RD may have certain therapeutic effects and pharmacological mechanisms in the process of organ fibrosis. However, there are no reports on the role and mechanism of RD in skin fibrosis. In this study, we used *in vitro* and *in vivo* experimental models to assess the antifibrotic properties and potential mechanisms of RD in skin fibrosis.

## 2. Materials and methods

### 2.1 Animals

All animal studys were conducted under the Institutional Animal Care and Use Committee (IACUC) of Nankai University (No. SYXK 2019‐0001). Animal work has taken place in the Nankai Animal Resources Center. Mice were anesthetized with Lidocaine hydrochloride (Sangon Biotech, China) and killed by CO_2_ asphyxiation.

C57BL/6J mice (male, 6–8 weeks) and nude BALB/c mice (female, 10 weeks) were obtained from Weitonglihua Company (Beijing, China). Animal housing condition was SPF level, 12 hours light/dark cycle, with free access to food and water.

### 2.2 Bleomycin‐induced skin fibrosis model

The skin fibrosis model was performed by daily injection bleomycin (BLM) (Blenoxane, Japan) for 3 weeks, while control group were given equal saline. BLM was dissolved in saline in concentration of 700μg/mL. As for treatment group, RD was dissolved in saline in concentration of 12.5 and 25μM, and was daily injected after treated with BLM. Mice were divided into four groups: NaCl group, mice were daily injected saline (100μL for each); BLM group, mice were singlely daily injected BLM solution (100μL for each); BLM+RD 12.5μM group, mice were daily injected both BLM solution (100μL for each) and 12.5μM RD (100μL for each); BLM+RD 25μM group, mice were daily injected both BLM solution (100μL for each) and 25μM RD (100μL for each).

### 2.3 Keloid xenograft mouse model

Briefly, the keloid tissues were weighed between 0.08 and 0.1g of each tissue block and sliced into sections. Anaesthesia was administered to the nude BALB/c mice before a 0.5cm cut was made on their backs, a pocket was created by severing the subcutaneous tissue, and a tissue block was inserted into the pocket. The model was established for approximately 2 weeks following the operation. Then, each graft was injected either RD (12.5, 25μM) or equal saline for during 14 days.

Keloid tissues were obtained from three patients during keloid removal surgery at the Tianjin Academy of Traditional Chinese Medicine Affiliated Hospital. All human participants signed informed consent before enrolling in the study, and all the regulations in this study were approved by the Ethics Committee of Nankai University on 20 December 2021 (approval No. NKUIRB2021116). The study was performed in accordance with the Declaration of Helsinki.

### 2.4 Histological examination

The skin samples were fixed in 4% paraformaldehyde, gradient alcohol and embedded in paraffin. Tissue sections were cut into 5μm thickness, then stained with hematoxylin and eosin (H&E), Masson’s trichrome, and Picrosirius red (Solarbio, Beijing, China). The pathological images of mice were randomly captured using transmission fluorescence microscope (Olympus, United States).

### 2.5 Hydroxyproline content determination

The skin was separated and then placed in ampoules with hydrochloric acid and dryed for 1 day at 120°C. The pH of the mixture was adjusted to 8.0, filtered, and then constant volume with PBS. Hydroxyproline (HYP) was conducted by a hydroxyproline detection kit, and the absorbance at 577nm was measured.

### 2.6 Immunohistochemical staining

Skin tissues were embedded in paraffin and cut into 5μm thickness. Then, the sections were incubated with antibodies including α‐SMA (Affinity, United States), SQSTM1/p62 (Affinity, United States), and p‐Smad3 (Cell Signaling Technology, United States). The pathological images were randomly photographed using transmission fluorescence microscope (Olympus, United States).

### 2.7 Isolation and cell culture of primary mouse fibroblasts

Primary mouse fibroblasts (PSFs) from newborn mice were isolated according to previously described methods [17]. Cells were cultured in DMEM with 10% FBS (Gibco, United States) and 1% penicillin and streptomycin (Gibco, United States). Cells were placed in cell incubator (Thermo, United States) at 37°C with 5% CO_2_.

### 2.8 Isolation and cell culture of keloid fibroblasts

Isolation and culture of keloid fibroblasts (KFs) were performed according to a previous protocol [18]. Keloid tissues, which was obtained from the patients, were divided into several fragments, seeded onto culture dishes. When tissues stick to the bottom of the dish, the medium was replaced with or without RD. Cells were cultured in DMEM with 10% FBS (Gibco, United States) and 1% penicillin and streptomycin (Gibco, United States). Representative images were acquired on day 9 after the KFs migrated from the edge of the tissues.

### 2.9 Cell counting Kit‐8 assay

The drug toxic and effect on PSFs and KFs were determined by Cell Counting Kit‐8 (CCK‐8) (Solarbio, China). A total of 10^3^ primary cultured PSFs and KFs were seeded in 96‐well plates per well and exposed to RD (12.5, 25μM). The cells were added with CCK‐8 solution at different days. The OD450nm was measured by a Microplate Reader 550 (Bio-Rad, USA).

### 2.10 EdU incorporation assay

Cell proliferation was tested with the EdU‐555 Imaging Kit (Beyotime Biotechnology, China). After treated with RD for 24h, EdU were added to PSFs for 2h, fixed with 4% paraformaldehyde. Then, a Click Additive Solution was added for 30min. Immunofluorescence images were gained by LSM800 microscope (Zeiss, Germany).

### 2.11 Wound‐healing assay

PSFs and KFs were cultured in a twelve‐well plate. Before treatment with TGF‐β1 (5ng/mL), PSFs were starved for 24h with serum-free medium. Then, RD was added with or without TGF‐β1 (5ng/mL) to PSFs. The scratch was captured at 0, 12, 24, 36, and 48h by an inverted optical microscope, and each group was imaged at three different locations.

### 2.12 Transwell assays

Transwell chambers (Corning, United States) were used to confirm the *in vitro* anti‐migratory and anti‐invasive effects of RD. For migration and invasion assays, the upper chamber was covered with or without Matrigel (BD Biosciences, United States). PSFs and KFs were cultured in the upper chamber with DMEM medium without serum, and various concentrations of RD were added. The lower chamber was cultured with DMEM containing 15% FBS (Gibco, United States) and the same concentration of RD as that in the upper compartment. After incubation for 24h, the migrated cells were incubated with 4% paraformaldehyde, stained with methylrosanilinium chloride solution (Beyotime Biotechnology, China), and imaged with a microscope.

### 2.13 Quantitative real‐time PCR

Total RNA was isolated with Trizol Reagent and reverse‐transcribed with FastKing gDNA SuperMix (TIANGEN Biotech, China). qRT‐PCR was conducted with SYBR Green Master Mix (Yeasen Biotech, China) based on the manufacturer’s protocols. The results were quantified with 2^-ΔΔCT^ method. GAPDH and β‐actin were served as internal reference in qRT-PCR. The sequences were listed in Table 1.

**Table 1:** Specific primers in qRT‐PCR analysis.

Gene	Primer	Sequence
M-β-actin	Forward	TGGATTTGGACGCATTGGTC
	Reverse	TTTGCACTGGTACGTGTTGAT
M-α-SMA	Forward	GCTGGTGATGATGCTCCCA
	Reverse	GCCCATTCCAACCATTACTCC
M-Col1α1	Forward	CCAAGAAGACATCCCTGAAGTCA
	Reverse	TGCACGTCATCGCACACA
M-Col1α2	Forward	GCAGGTTCACCTACTCTGTCCT
	Reverse	CTTGCCCCATTCATTTGTCT
M-Fn1	Forward	AAGGATGGAGTGATAGCAACCC
	Reverse	TCTGCTTGAAATCTGGTGTGC
M-IL-6	Forward	CTGCAAGAGACTTCCATCCAG
	Reverse	AGTGGTATAGACAGGTCTGTTGG
M-MMP-2	Forward	CAAGTTCCCCGGCGATGTC
	Reverse	TTCTGGTCAAGGTCACCTGTC
M-MMP-9	Forward	CTGGACAGCCAGACACTAAAG
	Reverse	CTCGCGGCAAGTCTTCAGAG
M-MMP-14	Forward	CAGTATGGCTACCTACCTCCAG
	Reverse	GCCTTGCCTGTCACTTGTAAA
M-LC3	Forward	TAGGCACCCACATAGGGTAT
	Reverse	CTACAACACCAGACCTGCTTA
M-P62	Forward	TGAAGGCTATTACAGCCAGAGTCAA
	Reverse	CCTTCAGTGATGGCCTGGT
H‐β‐actin	Forward	AGGCCAACCGTGAAAAGATG
	Reverse	AGAGCATAGCCCTCGTAGATGG
H-α-SMA	Forward	TGGGTGAACTCCATCGCTGTA
	Reverse	GTCGAATGCAACAAGGAAGCC
H-Col1α1	Forward	AAGCCGGAGGACAACCTTTTA
	Reverse	GCGAAGAGAATGACCAGATCC
H-Col3α1	Forward	TGGTGTTGGAGCCGCTGCCA
	Reverse	CTCAGCACTAGAATCTGTCC
H-Fn1	Forward	TGTCAGTCAAAGCAAGCCCG
	Reverse	TTAGGACGCTCATAAGTGTCACCC
H-LC3	Forward	CCGACCGCTGTAAGGAGGTA
	Reverse	TCACCCTTGTAGCGCTCGAT
H-P62	Forward	AGGATGGGGACTTGGTTGC
	Reverse	TCACAGATCACATTGGGGTGC

M, mouse; H, human.

### 2.14 Western blot

As mentioned above, proteins were collected from cells or skin tissues. SDS‒PAGE was used to separate the total protein samples, and the proteins were transferred to PVDF membranes. Following blocking, primary antibodies were used to investigate the expression of the proteins. Then, the membranes were incubated with HRP‐conjugated secondary antibodies and detected with an ECL system (Affinity Bioscience, United States). The primary antibodies are listed in Table 2.

**Table 2:** Specific primary antibodies in Western blot.

Antibody	Company	Country
α‐SMA Col-Ⅰ p‐Smad3 Smad3 p-pan-AKT pan-AKT p-PI3K p85 PI3K p85 p-mTOR mTOR LC3Ⅰ/Ⅱ SQSTM1/p62 Tubulin GAPDH	Affinity Cell Signaling Technology Cell Signaling Technology Cell Signaling Technology Affinity Affinity Affinity Affinity Cell Signaling Technology Cell Signaling Technology Cell Signaling Technology Affinity Affinity Affinity	United States United States United States United States United States United States United States United States United States United States United States United States United States United States

### 2.15 Immunofluorescence staining

PSFs and KFs were incubated with 4% paraformaldehyde for 20min, mixed with 0.2% Triton X‐100 for 10min, and then blocked with 5% BSA for 1h. PSFs and KFs were fixed with antibodies including SQSTM1/p62 (Affinity, United States). Then, cells were placed overnight at 4°C, followed by incubation with FITC‐conjugated secondary antibodies on the second day. Finally, the cells were stained with DAPI without light. Immunofluorescence images were gained by LSM800 microscope (Zeiss, Germany).

### 2.16 Statistical analysis

All statistical data were analyzed using GraphPad Prism 8.0 as Means ± SD. Differences between groups were assessed by one-way ANOVA with Tukey’s post hoc multiple comparison tests. *P* < 0.05 was considered of statistical significance.

## 3. Results

### 3.1 RD ameliorates BLM-induced skin fibrosis *in vivo*

**Fig 1:**
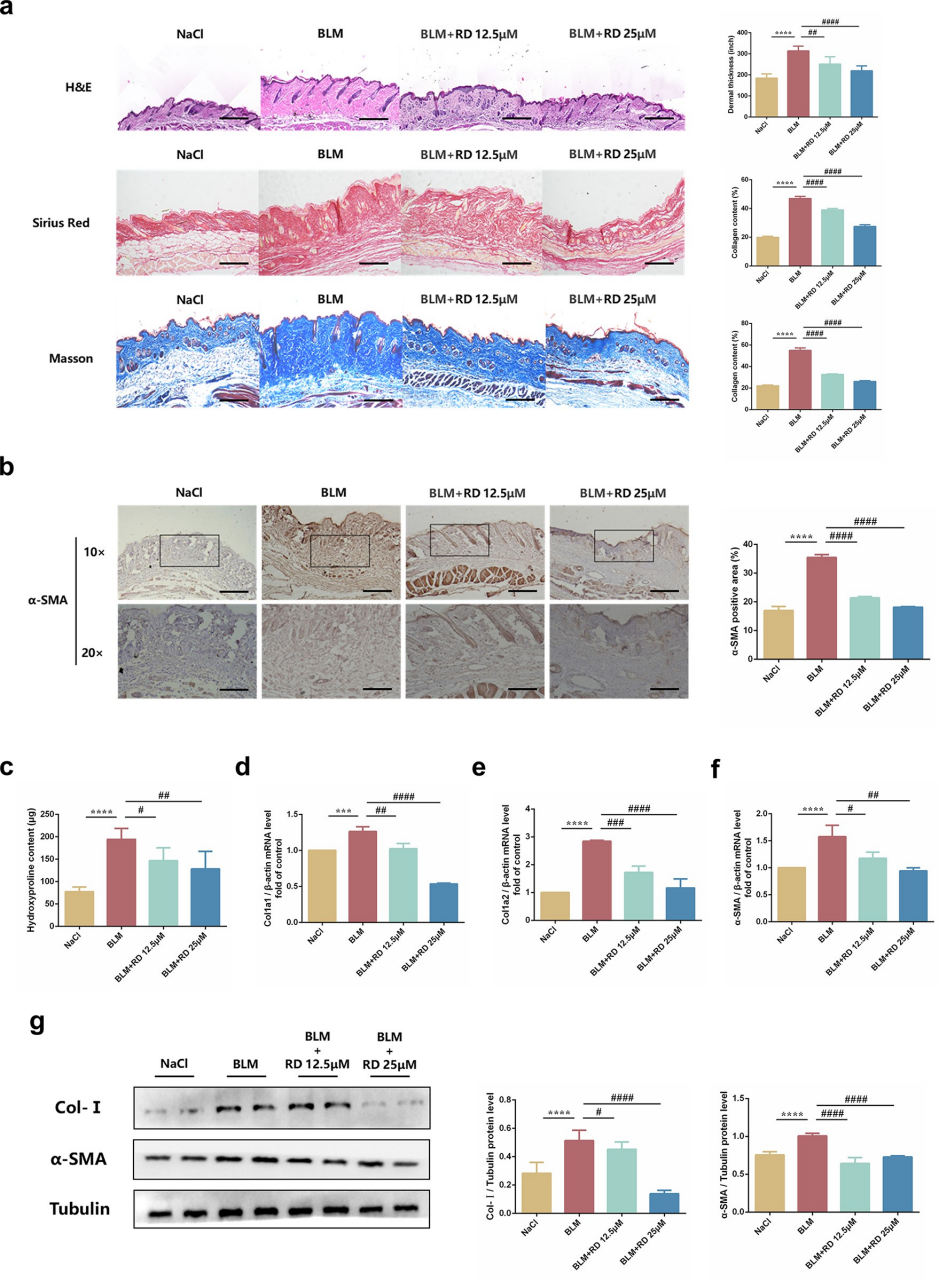
RD ameliorates BLM‐induced skin fibrosis *in vivo*. (a) Representative skin sections stained with hematoxylin–eosin (H&E), Sirius red and Masson’s trichrome staining (20×, Scale bar = 50μm) (n = 6). Total dermal thickness of the back of each group of mice based on Hematoxylin–eosin (H&E) images. Collagen density was quantified on Masson’s trichrome and Sirius red images. (b) Immunohistochemical staining analysis and of α-SMA in the lesional skin of each group (10× and 20×, Scale bar = 50μm) (n = 6). (c) Hydroxyproline content of skin tissues in C57BL/6J mice (n = 6). (d) The mRNA levels of Col1α1 in the lesional skin (n = 6). (e) The mRNA levels of Col1α2 in the lesional skin (n = 6). (f) The mRNA levels of α‐SMA in the lesional skin (n = 6). (g) The protein levels of Col-Ⅰ and α‐SMA in the lesional skin (n = 6). The data are shown as mean ± SD (one-way ANOVA with Tukey’s post-hoc multiple comparison tests). ***, p < 0.001, ****, p < 0.0001 vs. NaCl; **#**, p < 0.05, **##**, p < 0.01, **###**, p < 0.001, **####**, p < 0.0001 vs. BLM. BLM, Bleomycin; RD, Remdesivir.

Since RD greatly ameliorates BLM-induced pulmonary fibrosis, we further investigated whether RD had effect on BLM-induced skin fibrosis. As expected, skin thickness and collagen deposition were increased in BLM-induced skin fibrosis mice but decreased in mice treated with RD **(Fig 1A)**. Meanwhile, the hydroxyproline content was increased in BLM-induced skin fibrosis mice but decreased in mice treated with RD **(Fig 1C)**. Fibroblasts activation is an important component of skin fibrosis, we then verified the regulatory effects of RD on fibroblast activation-related marker. Consistently, the fibrotic gene Collagen I (Col-Ⅰ), Fibronectin (Fn) and α-Smooth muscle actin (α-SMA) were increased in BLM-induced skin fibrosis tissues, while decreased in tissues treated with RD **(Fig 1D–1F)**. Similarly, the protein levels of Col-Ⅰ and α-SMA showed the same results **(Fig 1B and 1G)**. Besides, matrix metalloproteinases (MMPs) are also involved in skin fibrosis. Interestingly, the mRNA levels of IL-6, MMP-2, MMP-9 and MMP-14 were increased in BLM-induced skin fibrosis mice, while decreased treated with RD **(S1 Fig)**. Taken together, these data demonstrated that RD could ameliorate BLM-induced skin fibrosis.

### 3.2 RD alleviates keloid xenografts-induced skin fibrosis *in vivo*

**Fig 2:**
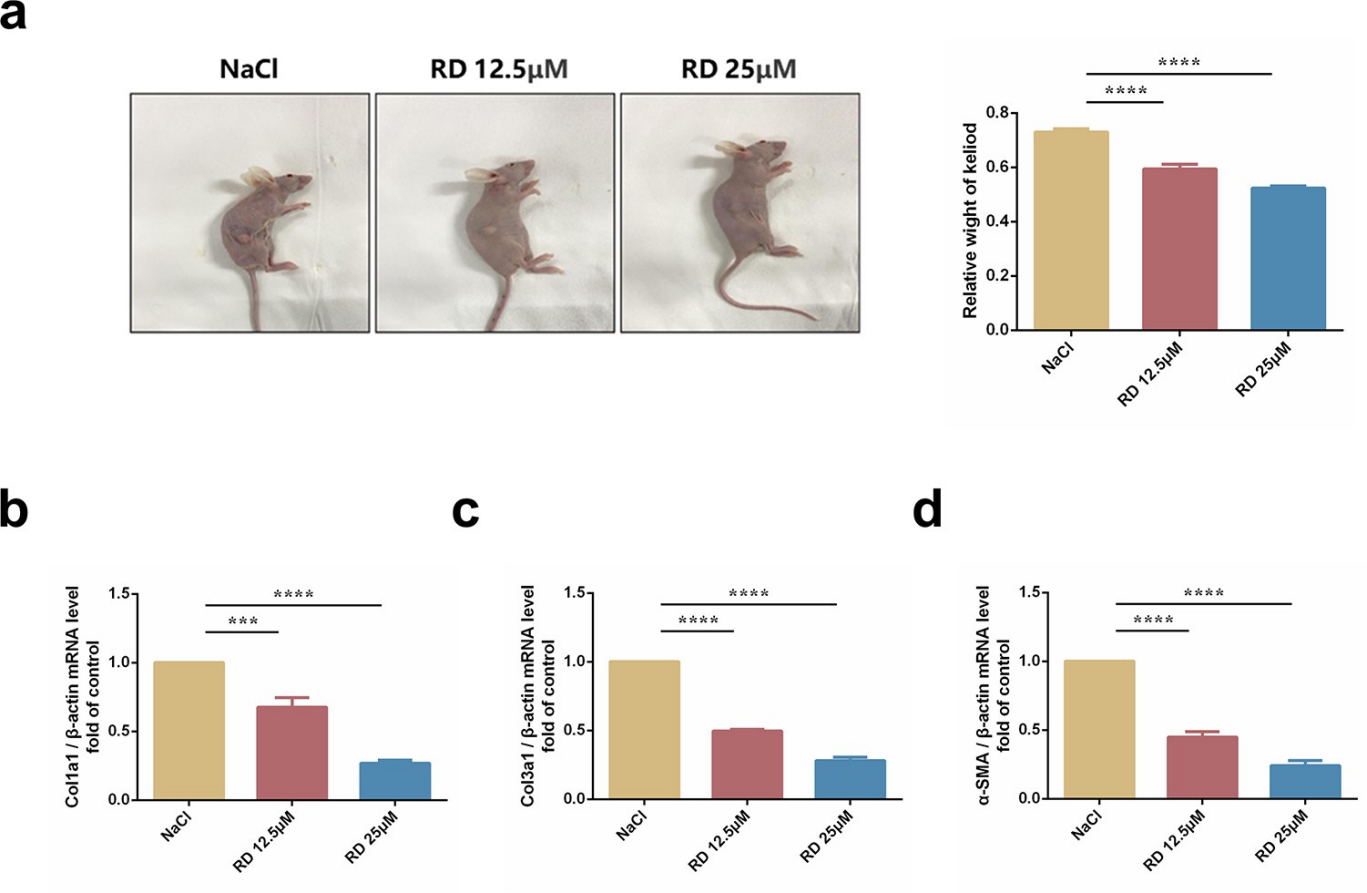
RD alleviates keloid xenografts-induced skin fibrosis *in vivo*. (a) Macrographic images and weight of xenografted tissues on the back of nude BALB/c mice after intralesional injection of RD (n = 3). (b) The mRNA levels of Col1α1 in xenografted keloid tissues (n = 3). (c) The mRNA levels of Col3α1 in xenografted keloid tissues (n = 3). (d) The mRNA levels of α‐SMA in xenografted keloid tissues (n = 3). The data are shown as mean ± SD (one-way ANOVA with Tukey’s post-hoc multiple comparison tests). ***, p < 0.001, ****, p < 0.0001 vs. NaCl. BLM, Bleomycin; RD, Remdesivir.

Interestingly, in a transplanted keloid xenograft nude mouse model, RD suppressed the weight of keloid tissues **(Fig 2A)**. Consistently, Col-Ⅰ, α-SMA and Fn were increased in keloid xenograft-induced skin fibrosis tissues, while decreased in tissues treated with RD **(Fig 2B–2E)**. Taken together, these data indicated that RD could alleviate xenografts-induced skin fibrosis.

### 3.3 RD alleviates mouse primary skin fibroblast and xenografted keloid fibroblast autophagy *in vivo*

**Fig 3:**
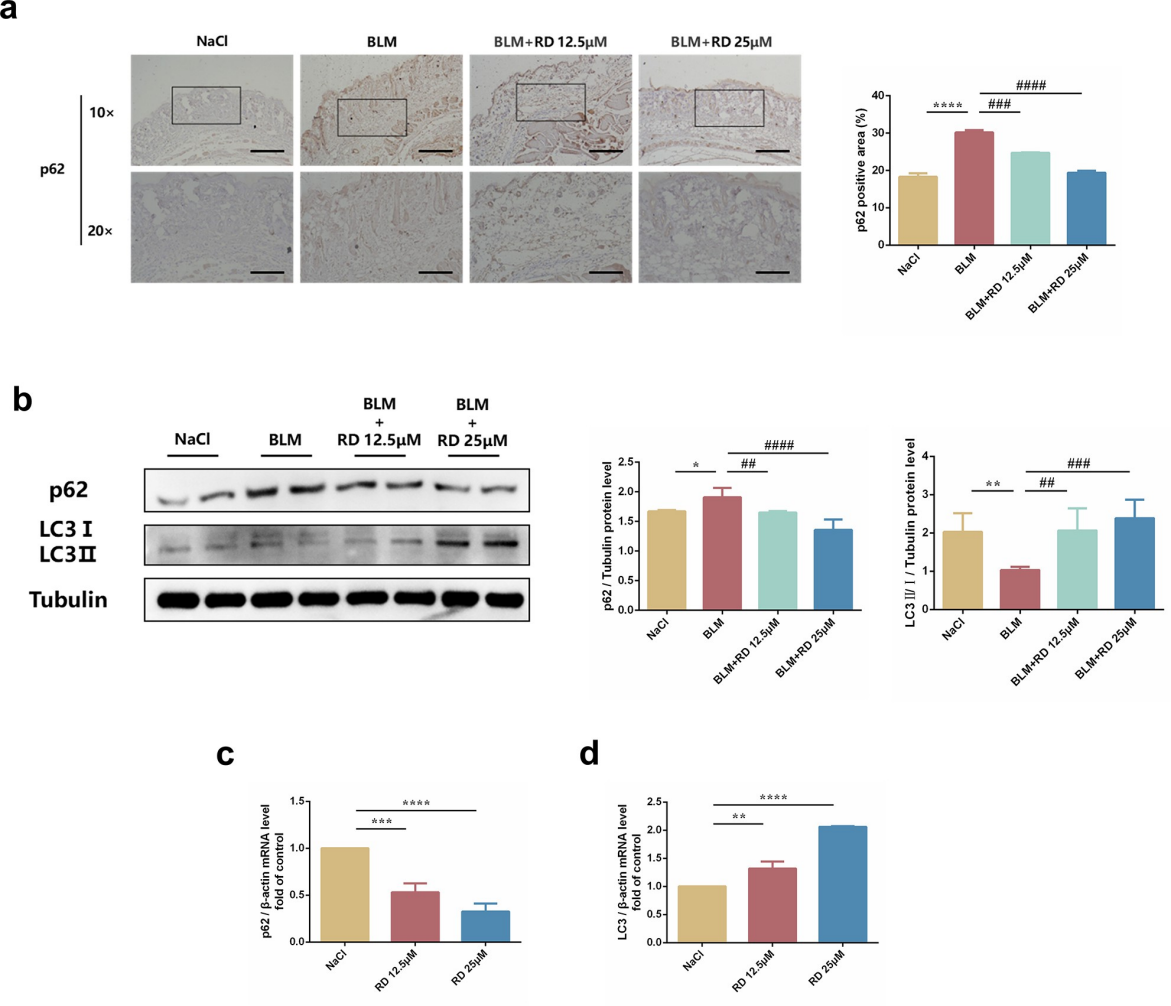
RD alleviates mouse primary skin fibroblasts and xenografted keloid fibroblasts autophagy *in vivo*. (a) Immunofluorescence staining of p62 in skin frozen sections of BLM‐induced model (10× and 20×, Scale bar = 50μm) (n = 6). (b) The protein levels of p62 and LC3 in the lesional skin (n = 6). (c) The mRNA levels of p62 in xenografted keloid tissues (n = 3). (d) The mRNA levels of LC3 in xenografted keloid tissues (n = 3). The data are shown as mean ± SD (one-way ANOVA with Tukey’s post-hoc multiple comparison tests). *, p < 0.05, **, p < 0.01, ***, p < 0.001, ****, p < 0.0001 vs. NaCl; **##**, p < 0.01, **###**, p < 0.001, **####**, p < 0.0001 vs. BLM. BLM, Bleomycin; RD, Remdesivir.

Autophagy is a physiological process that maintains cell homeostasis and is also closely related to organ fibrosis. Therefore, we further explored whether RD had effect on fibroblast autophagy. Interestingly, autophagy-related proteins p62 and LC3 were increased in BLM-induced skin fibrosis tissues but decreased in tissues treated with RD **(Fig 3A and 3B)**. Consistently, in the transplanted keloid xenograft nude mouse model, RD showed the same results **(Fig 3C and 3D)**. Taken together, these data showed that RD could alleviate PSF and KF autophagy *in vivo*.

### 3.4 RD attenuates TGF-β1-induced mouse primary skin fibroblast activation *in vitro*

**Fig 4:**
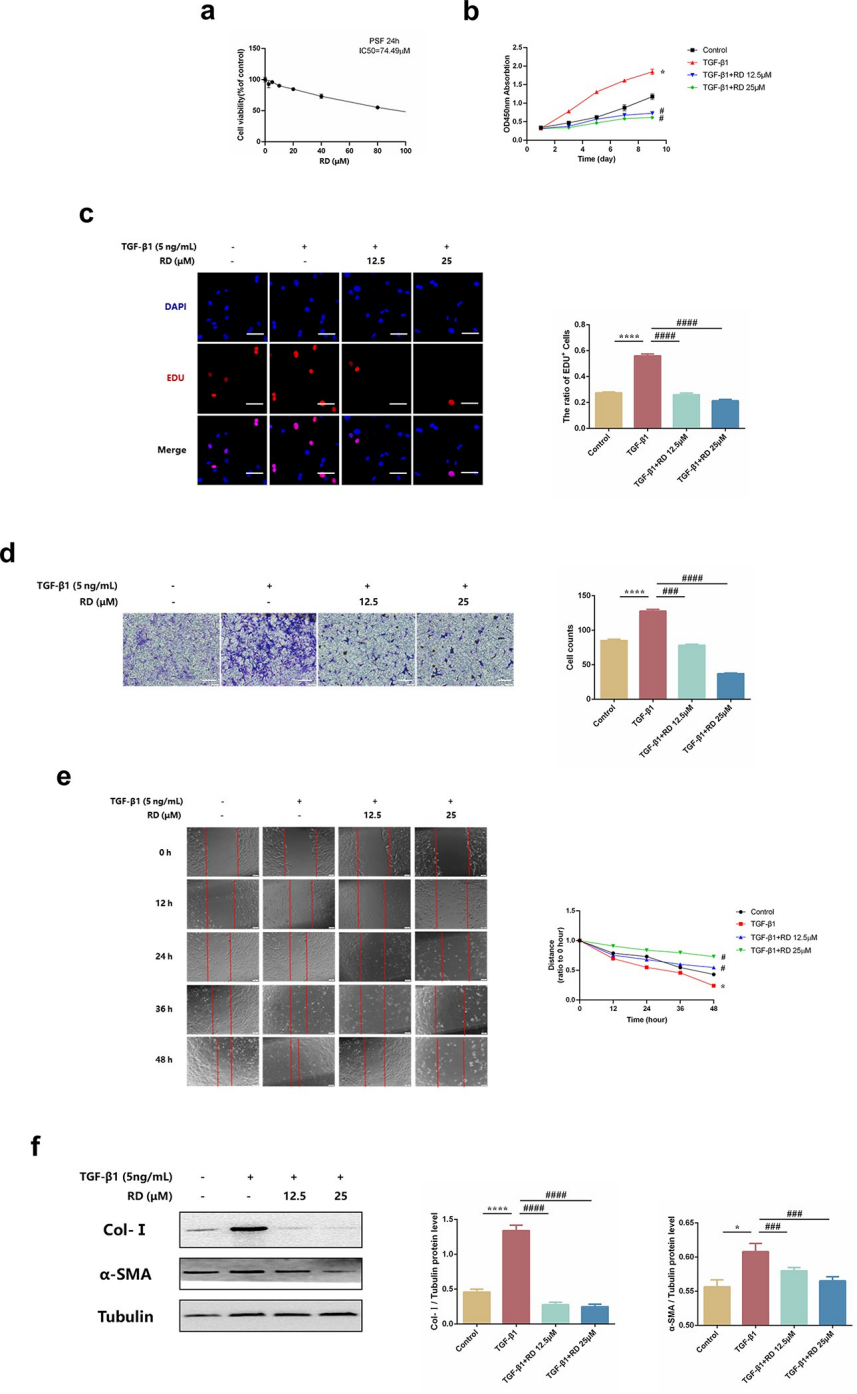
RD attenuates TGF-β1-induced mouse primary skin fibroblasts activation *in vitro*. (a) CCK-8 assays of PSFs toxic (n = 6). Cells were exposed to the indicated doses of RD (0 to 80μM) for 24 h, IC50 = 74.49μM. (b) CCK-8 assays of PSFs proliferation (n = 6). Cells were performed to test the effect of RD on cell proliferation of TGF-β1-stimulated PSFs. (c) Representative images and quantitative results of EdU incorporation assay in PSFs (n = 6). The ratio of EdU‐positive cells to DAPI‐labeled cells in each group was determined (×40, Scale bar = 100μm). (d) Representative images and quantitative results of migration in PSFs (n = 6). Representative images were captured and counted under a fluorescence microscope at ×20 (scale bar = 100μm). (e) Representative images and quantitative analysis of wound healing assay in PSFs (n = 3). The wound closure was captured at 0, 12, 24, 36, and 48h after scratch generation. (f) The protein levels of Col-Ⅰ and α-SMA in PSFs (n = 3). PSFs were treated with RD (12.5, 25μM) and TGF-β1 (5ng/ml) for 24h. The data are shown as mean ± SD (one-way ANOVA with Tukey’s post-hoc multiple comparison tests). *, p < 0.05, ***, p < 0.001, ****, p < 0.0001 vs. Control; **#**, p < 0.05, **##**, p < 0.01, **###**, p < 0.001, **####**, p < 0.0001 vs. TGF-β1. RD, Remdesivir.

We soon detected whether RD had regulatory effects on fibroblast activation *in vitro*. We first explored the appropriate dose of RD in PSFs. The results demonstrated that 12.5 and 25μM were safe doses in PSFs **(Fig 4A)**. Since fibroblast activation mainly includes fibroblast proliferation, migration, transdifferentiation and ECM synthesis in skin fibrosis, we further evaluated the effect of RD on TGF‐β1-induced PSF proliferation. Interestingly, cell proliferation was increased in TGF-β1-induced PSFs but decreased in those treated with RD **(Fig 4B)**. Meanwhile, EdU staining of PSFs demonstrated the same results **(Fig 4C)**. Furthermore, Transwell and wound healing experiments also showed a significant inhibitory effect of RD on the migration of TGF‐β1‐induced PSFs **(Fig 4D and 4E)**. In addition, the levels of fibrotic marker Col-Ⅰ and α‐SMA were increased in TGF-β1-induced PSFs but decreased in those treated with RD **(Fig 4F)**. Taken together, these data suggested that RD could attenuate TGF-β1-induced PSF activation *in vitro*.

### 3.5 RD attenuates keloid fibroblast activation *in vitro*

**Fig 5:**
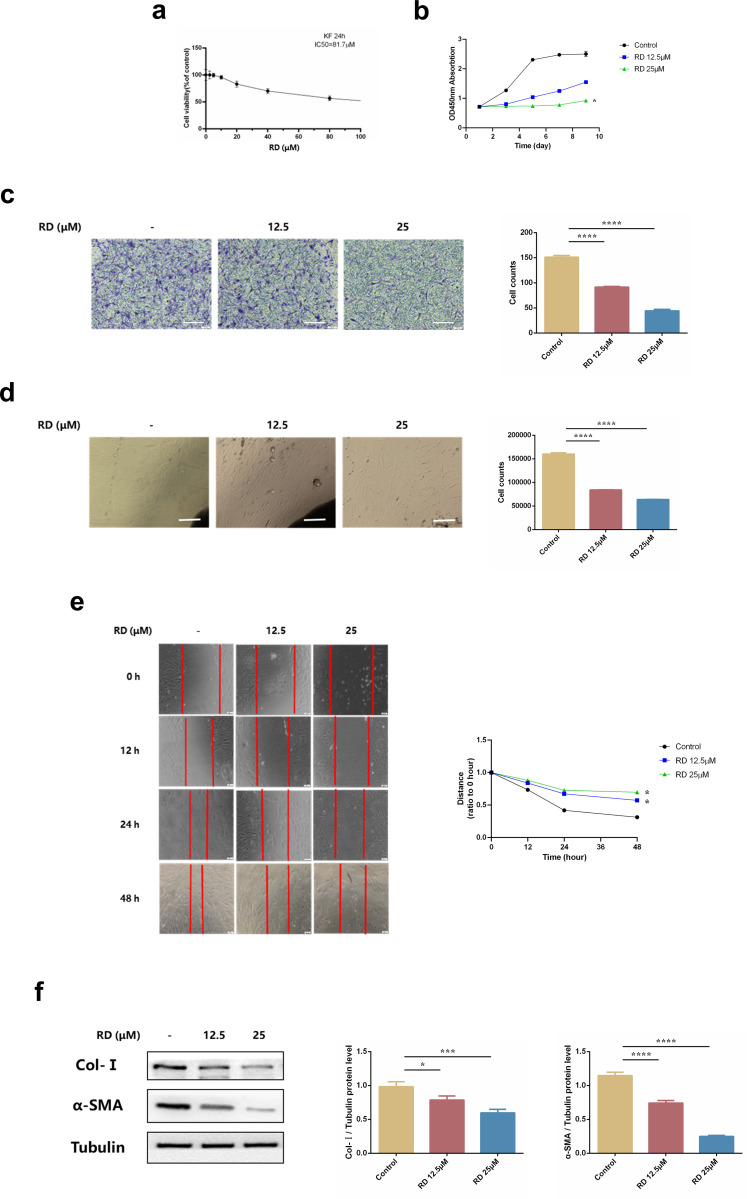
RD attenuates keloid fibroblasts activation *in vitro*. (a) CCK-8 assays of KFs toxic (n = 6). Cells were exposed to the indicated doses of RD (0 to 80μM) for 24h, IC50 = 81.7μM. (b) CCK-8 assays of KFs proliferation (n = 6). Cells were performed to test the effect of Remdesivir on cell proliferation of TGF-β1-stimulated KFs. (c) Representative images and quantitative results of migration in KFs (n = 6). Representative images were captured and counted under a fluorescence microscope at ×20 (Scale bar = 100μm). (d) Representative images of tissue explants of KFs (n = 3). KFs were cultured with RD (12.5, 25μM) at day 9 (×40, scale bar = 200μm). The cell numbers that migrated out of the tissue explants were quantified at day 9. (e) Representative images and quantitative analysis of wound healing assay in KFs (n = 3). The wound closure was captured at 0, 12, 24 and 48h after scratch generation. (f) The protein levels of Col-Ⅰ and α-SMA in KFs (n = 3). KFs were treated with RD (12.5, 25μM) for 24 h. The data are shown as mean ± SD (one-way ANOVA with Tukey’s post-hoc multiple comparison tests). *, p < 0.05, ***, p < 0.001, ****, p < 0.0001 vs. Control. RD, Remdesivir.

As mentioned above, we then explored the appropriate dose of RD in KFs. The results demonstrated that 12.5 and 25μM were safe doses in KFs **(Fig 5A)**. Similarly, cell proliferation was decreased after treatment with RD **(Fig 5B)**. Furthermore, Transwell, wound healing, and scratch experiments also showed a significant inhibitory effect of RD on the migration and invasion of KFs **(Fig 5C–5E)**. In addition, the levels of the transdifferentiation marker α‐SMA and the ECM synthesis marker Col-Ⅰ were decreased in KFs treated with RD **(Fig 5F)**. Taken together, these data suggested that RD could attenuate KF activation *in vitro*.

### 3.6 RD suppresses fibroblast activation via TGF-β/Smad signaling pathway

**Fig 6:**
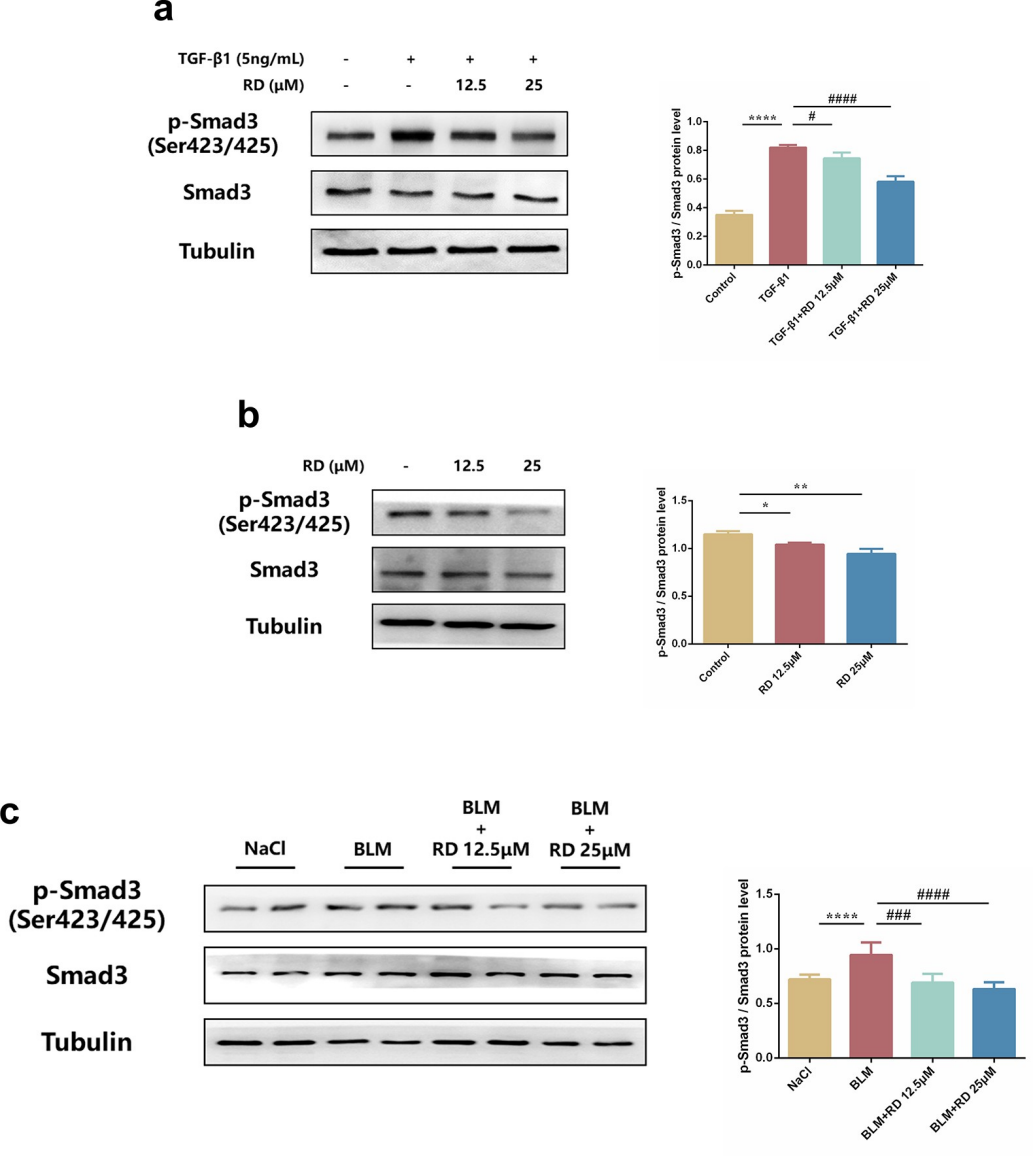
RD suppresses TGF-β1/Smad signaling pathway both *in vivo* and *in vitro*. (a) The protein levels of phosphorylated Smad3 in PSFs (n = 3). (b) The protein levels of phosphorylated Smad3 in KFs (n = 3). (c) The protein levels of phosphorylated Smad3 in mice skin tissues of each group (n = 6). PSFs were treated with TGF-β1 (5ng/mL) and RD (12.5, 25μM) for 24h. KFs were treated with RD (12.5, 25μM) for 24h. The data are shown as mean ± SD (one-way ANOVA with Tukey’s post-hoc multiple comparison tests). *, p < 0.05, **, p < 0.01, ****, p < 0.0001 vs. Control or NaCl. **#**, p < 0.05, **##**, p < 0.01, **###**, p < 0.001, **####**, p < 0.0001 vs. TGF-β1 or BLM. BLM, Bleomycin; RD, Remdesivir.

We further explored the underlying mechanism of RD on fibroblast activation. Since the TGF-β/Smad signaling pathway is one of the most classical signaling pathways in fibroblast activation, we then investigated the TGF-β/Smad signaling pathway in fibroblast activation. As expected, the levels of phosphorylated Smad3 were increased in TGF-β1-induced PSFs while decreased in those treated with RD **(Fig 6A)**. Similarly, the levels of phosphorylated Smad3 were decreased in KFs treated with RD **(Fig 6B)**. In addition, RD suggested the same results in skin fibrosis tissues **(Fig 6C)**. Taken together, these data demonstrated that RD could suppress fibroblast activation by TGF-β1/Smad3 both *in vivo* and *in vitro*.

### 3.7 RD attenuates mouse primary skin fibroblast and keloid fibroblast autophagy *in vitro*

**Fig 7:**
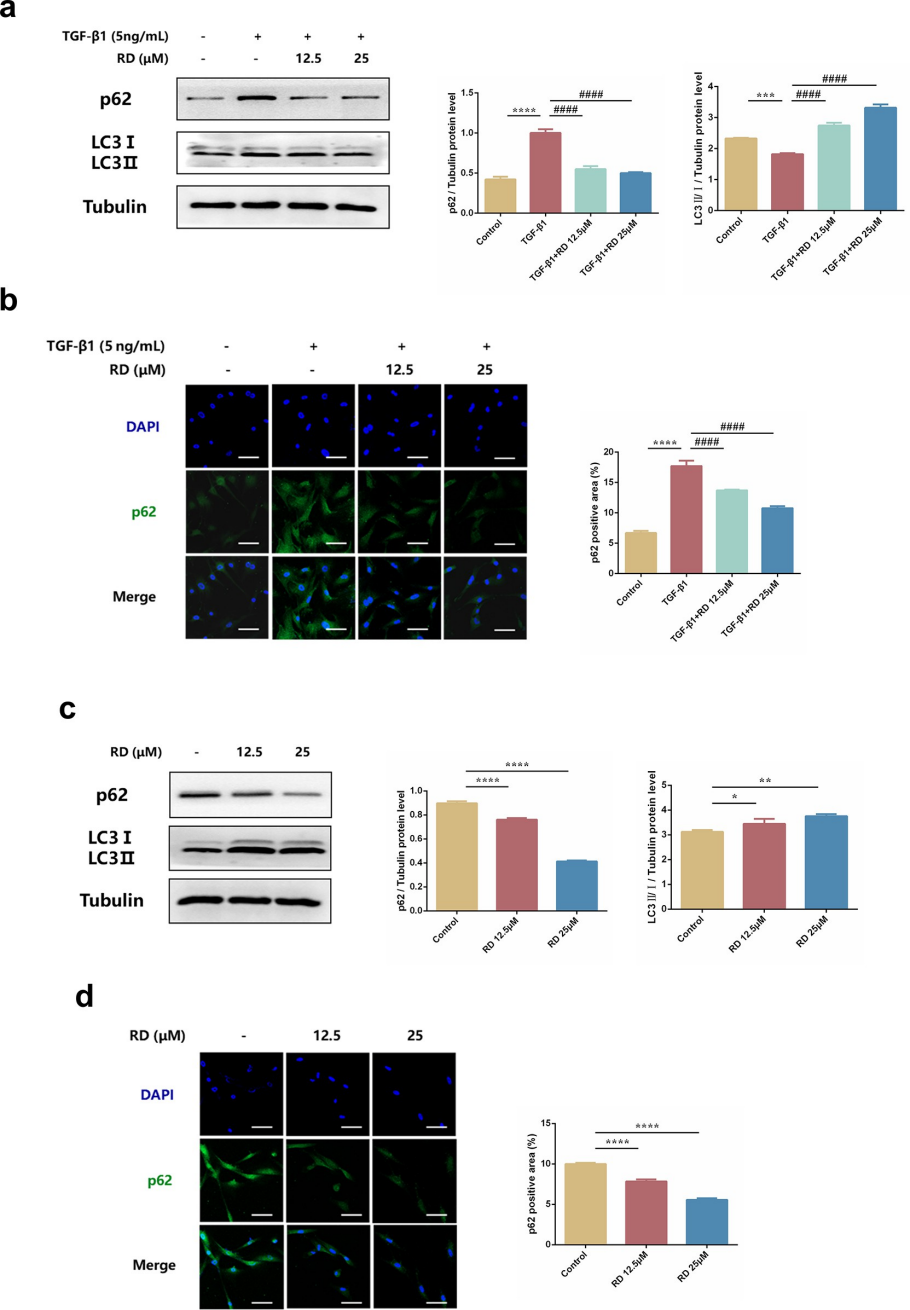
RD attenuates mouse primary skin fibroblasts and keloid fibroblasts autophagy *in vitro*. (a) The protein levels of p62 and LC3 in PSFs (n = 3). (b) Immunofluorescence staining of p62 in PSFs (n = 3). (c) The protein levels of p62 and LC3 in KFs (n = 3). (d) Immunofluorescence staining of p62 in KFs (n = 3). PSFs were treated with TGF-β1 (5ng/mL) and RD (12.5, 25μM). KFs were treated with RD (12.5, 25 μM) for 24h. The data are shown as mean ± SD (one-way ANOVA with Tukey’s post-hoc multiple comparison tests). *, p < 0.05, **, p < 0.01, ***, p < 0.001, ****, p < 0.0001 vs. Control; **####**, p < 0.0001 vs. TGF-β1. RD, Remdesivir.

We then explored whether RD had regulatory effects on fibroblast autophagy *in vitro*. Interestingly, the autophagy-related proteins p62 and LC3-II/I ratio were increased in TGF-β1-induced PSFs while decreased in those treated with RD **(Fig 7A and 7B)**. Consistently, in KFs, RD showed the same results **(Fig 7C and 7D)**. Taken together, these data demonstrated that RD could attenuate PSF and KF autophagy *in vitro*.

### 3.8 RD suppresses fibroblast autophagy via PI3K/Akt/mTOR signaling pathway

**Fig 8:**
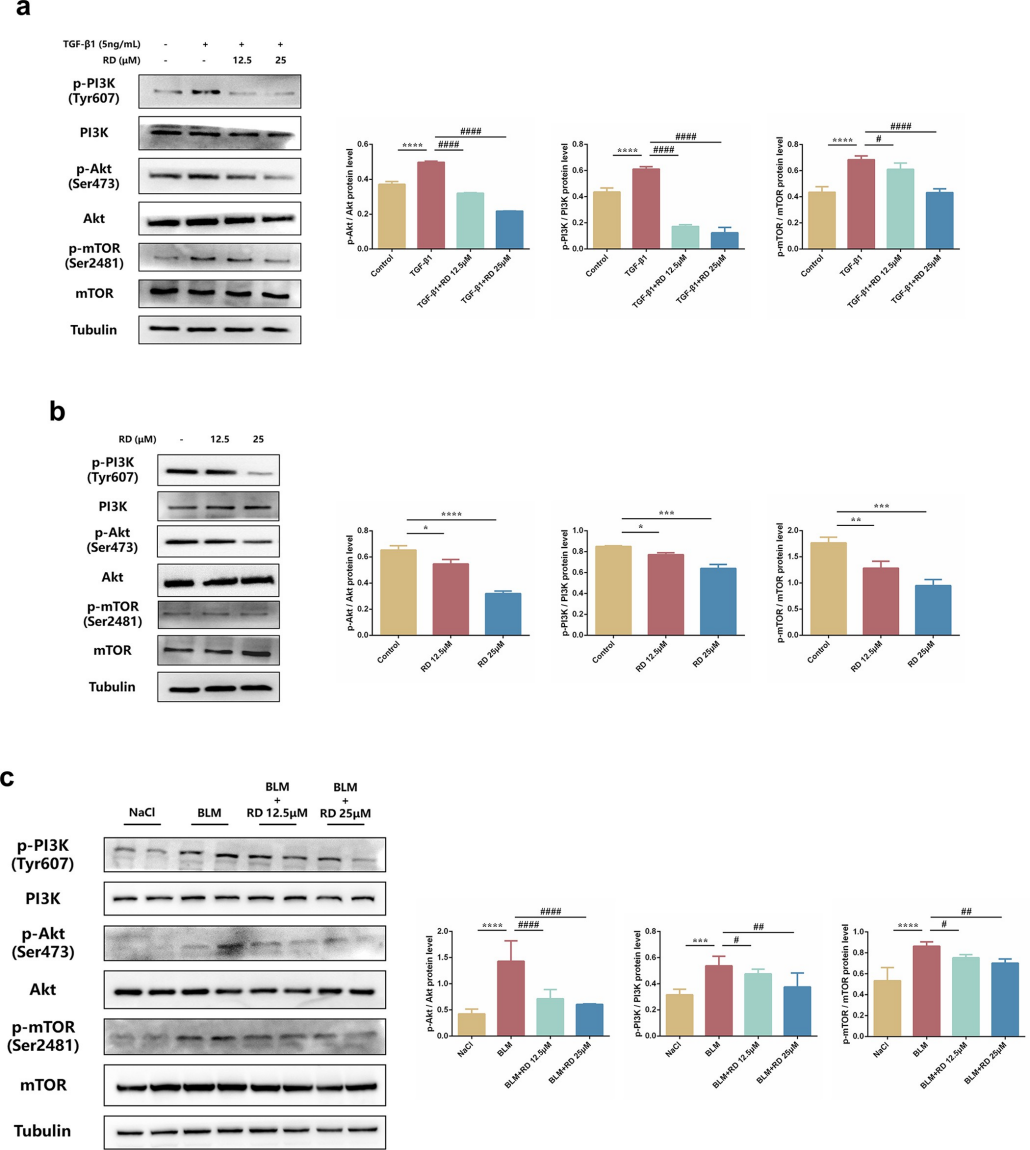
RD suppresses autophagy by PI3K/Akt/mTOR signaling pathway both *in vivo* and *in vitro*. (a) The protein levels of phosphorylated Akt, PI3K and mTOR in PSFs (n = 3). (b) The protein levels of phosphorylated Akt, PI3K and mTOR in KFs (n = 3). (c) The protein levels of phosphorylated Akt, PI3K and mTOR in mice pathogenic skin tissues of each group (n = 6). PSFs were treated with TGF-β1 (5 ng/mL) and RD (12.5, 25μM) for 24h. KFs were treated with RD (12.5, 25 μM) for 24h. The data are shown as mean ± SD (one-way ANOVA with Tukey’s post-hoc multiple comparison tests). *, p < 0.05, **, p < 0.01, ***, p < 0.001, ****, p < 0.0001 vs. Control or NaCl. **##**, p < 0.01, **####**, p < 0.0001 vs. TGF-β1 or BLM. BLM, Bleomycin; RD, Remdesivir.

We further explored the underlying mechanism of RD on fibroblast autophagy. Since the PI3K/Akt/mTOR signaling pathway is a key signaling pathway and is excessive expressed in fibroblast autophagy. As expected, the levels of phosphorylated PI3K, Akt, and mTOR were increased in TGF-β1-induced PSFs but decreased in those treated with RD **(Fig 8A)**. Similarly, the levels of phosphorylated PI3K, Akt, and mTOR were decreased in KFs treated with RD **(Fig 8B)**. In addition, RD suggested the same results in skin fibrosis tissues **(Fig 8C)**. Taken together, these data demonstrated that RD could attenuate autophagy by PI3K/Akt/mTOR both *in vivo* and *in vitro*.

### 3.9 Mechanism for the anti-skin fibrosis effect of RD

**Fig 9:**
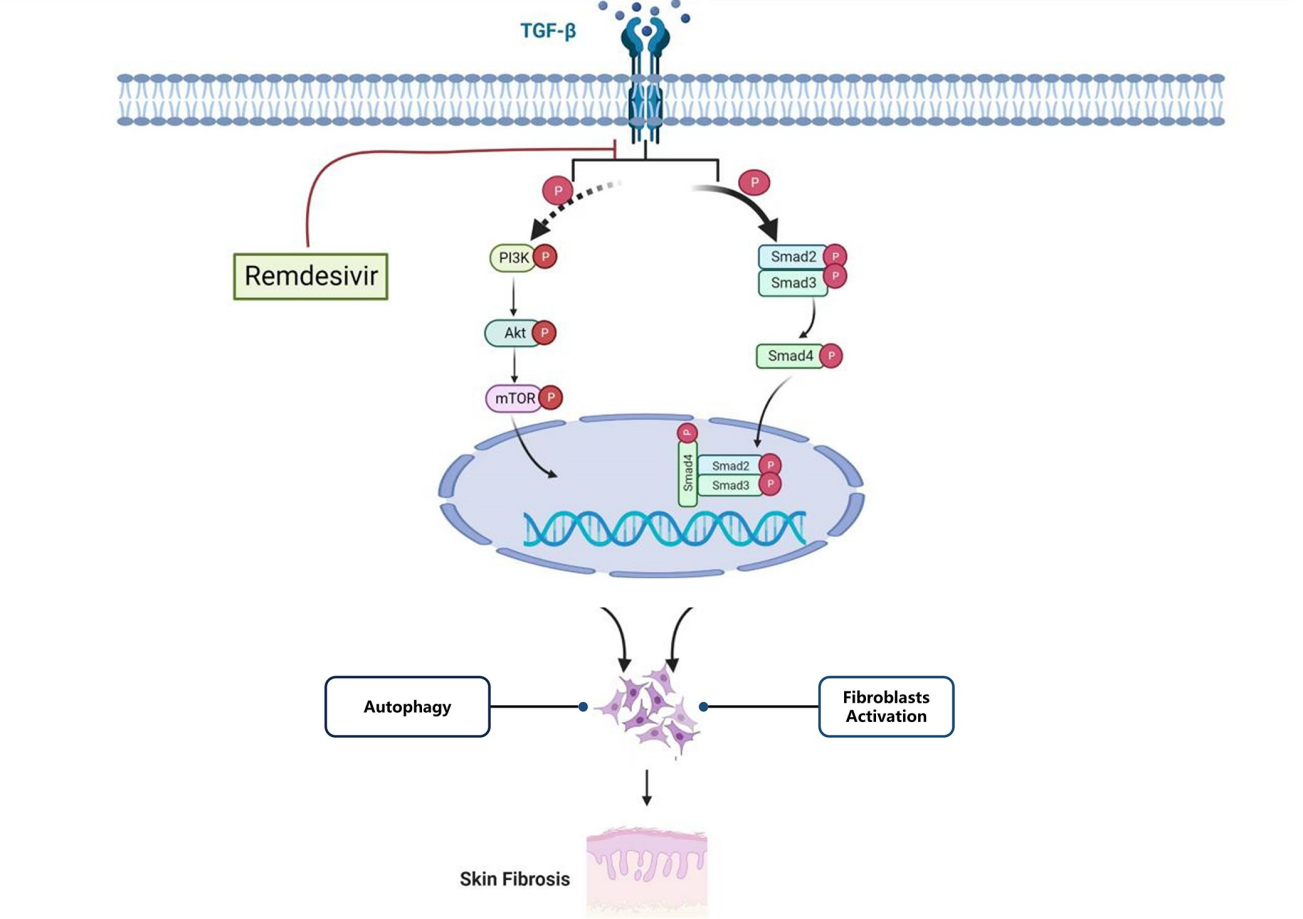
Mechanism for the anti-skin fibrosis effect of RD.

In summary, our study presented suggested that RD effectively alleviate skin fibrosis in mice model and keloid xenografts. The mechanism studies indicated that RD could suppress skin fibroblasts activation by TGF‐β1/Smad signaling and inhibit skin fibroblast autophagy by PI3K/Akt/mTOR signaling **(Fig 9)**. Based on the above results, RD may serve as an anti‐fibrotic drug candidate in skin fibrosis treatment. Therefore, our present study was of benefit to provide a novel strategy for the mechanism exploration and treatment of keloid and some other fibrotic diseases.

## 4. Discussion

Skin fibrosis, which is defined by excessive skin fibroblasts proliferation and extracellular matrix deposition, is a histopathological characteristic of dermatologic illness [19]. Keloid, caused by skin fibroblasts of abnormal growth, is a kind of pathological scar disease. The readily available drugs and methods for treating pathological scars are limited mainly due to incomplete understanding of the mechanism of scar formation.

During the process of keloid formation, the function of many types of cell populations abnormally changes [20,21]. Fibroblasts, the primary effector cells in keloids, eventually lead to keloid formation by inducing a persistent inflammatory response and excessive extracellular matrix (ECM) deposition [22]. At the same time, TGF-β signaling pathway in skin fibroblasts is of great activation, which modulates skin fibrosis. Although RD is generally acknowledged as an antiviral drug, recent studies have proven that RD can attenuate various organs fibrosis in, such as kidneys and lungs. Thus, we detected the effects and mechanisms on skin fibrosis. Our findings suggested that RD inhibited the proliferation, migration and activation of skin fibroblasts and reduced the deposition of ΕCM *in vitro*. At the same time, *in vivo* experiments showed that RD alleviated BLM‐induced skin fibrosis in mice, inhibited fibrogenic activation, and reduced the weight and ECM gene expression of xenografted keloid tissues. In the mechanistic study, we found that RD restrained transdifferentiation via TGF-β-dependent pathway.

The existing pathogenesis research shows that excessive skin fibrosis is caused by lots of factors, including a chronic inflammatory state, inadequate autophagy-mediated fibroblasts transdifferentiation [23]. According to previous studies, the autophagic flux in fibroblasts from keloid patients seems to be impaired when compared with that in normal cells [24]. It is well known that autophagy is induced by the PI3K/Akt/mTOR signaling pathways, and when these signaling pathways are activated, autophagy is inhibited [25]. In addition, inhibition of PI3K/Akt signaling provides protection against keloids by inhibiting collagen synthesis and excessive proliferation in keloid fibroblasts. In this study, we demonstrated that RD promoted autophagy in skin fibroblasts by inhibiting the PI3K/Akt/mTOR pathway. Although the center mechanisms have not been thoroughly researched, this funding can help uncover the underlying mechanisms of skin scarring and provide potential therapeutic targets for regenerative treatment of skin injuries.

For scar therapy, understanding the pathological scar mechanism is extremely important. Animal scar models are one of the crucial techniques used today to examine abnormal scars. In terms of histology, cytology, and other factors, the ideal animal model of scar should be as similar with the human pathological scar as possible [26]. In this study, we established a keloid xenotransplantation model in nude mice to simulate human pathological scars [27,28]. The advantage of this model is that scar skin can maintain the physiological characteristics of human skin. However, it easily infects diseases and leads to a reduction in life span, and it is difficult for the defective immune system to imitate the normal microenvironment of the body [29]. According to our study, intralesional injection of RD can reduce ECM gene expression in xenografted keloid tissue, which agrees with the outcomes of the *in vitro* and animal model studies.

## Supporting information

S1 Fig Supplemental data. (DOC)

S1 File Original data. (DOCX)
