# Supplementary material for: Remdesivir alleviates skin fibrosis by suppressing TGF-β1 signaling pathway
Source: PLoS One. 2024 Jul 18;19(7):e0305927. doi: 10.1371/journal.pone.0305927 (PMC11257276; doi:10.1371/journal.pone.0305927)
Supplement: S1 Fig — (DOC) [file pone.0305927.s001.doc]

**Figure S1**

**
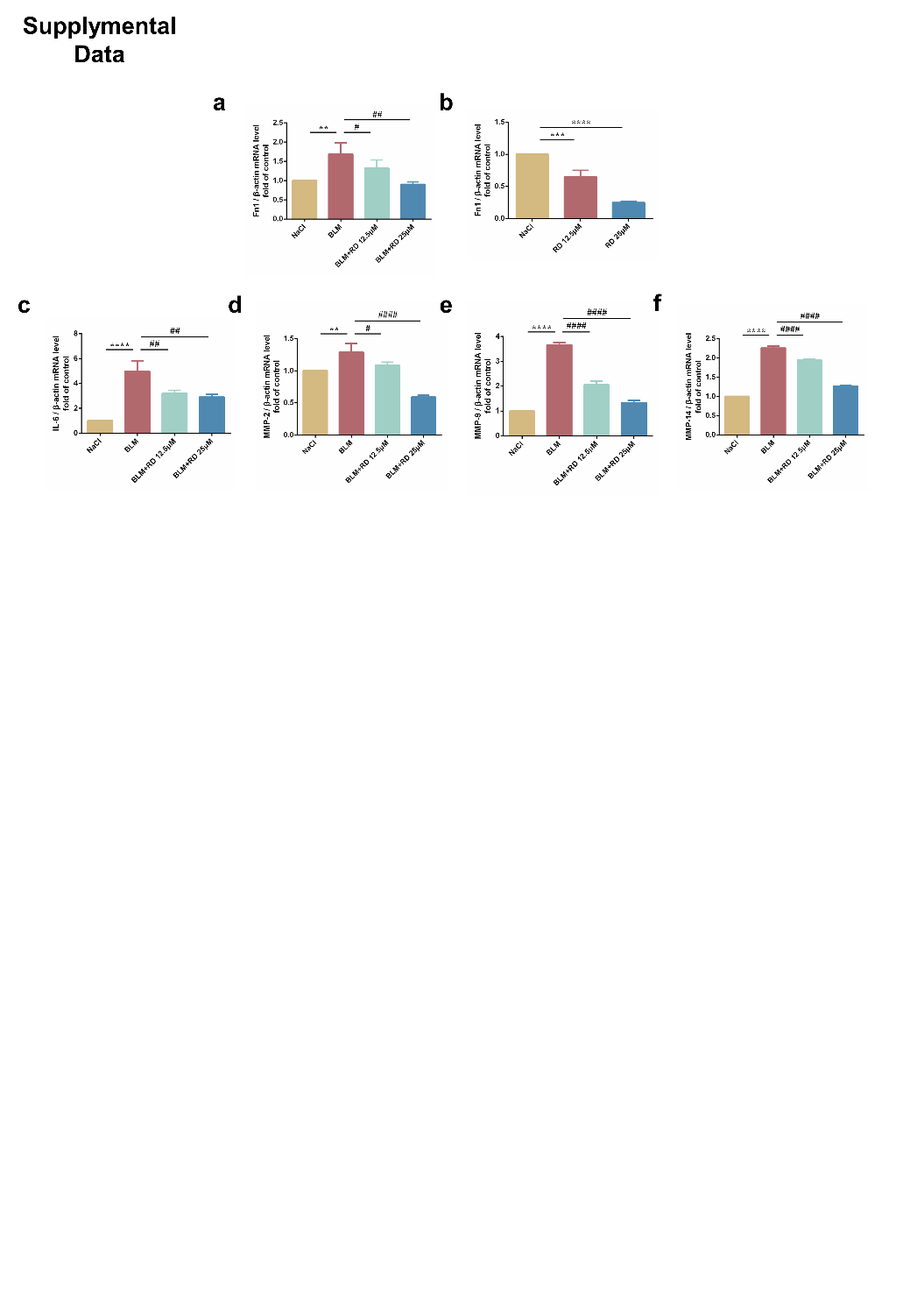
**

(a) The mRNA levels of Fn1 in the lesional skin (n=6). (b) The mRNA levels of Fn1 in xenografted keloid tissues (n=3). (c) The mRNA levels of IL-6 in the lesional skin (n=6). (d) The mRNA levels of MMP-2 in the lesional skin (n=6). (e) The mRNA levels of MMP-9 in the lesional skin (n=6). (f) The mRNA levels of MMP-14 in the lesional skin (n=6).
