# Supplementary material for: Remdesivir alleviates skin fibrosis by suppressing TGF-β1 signaling pathway
Source: PLoS One. 2024 Jul 18;19(7):e0305927. doi: 10.1371/journal.pone.0305927 (PMC11257276; doi:10.1371/journal.pone.0305927)
Supplement: S1 File — (DOCX) [file pone.0305927.s002.docx]

**Supplementary Material**


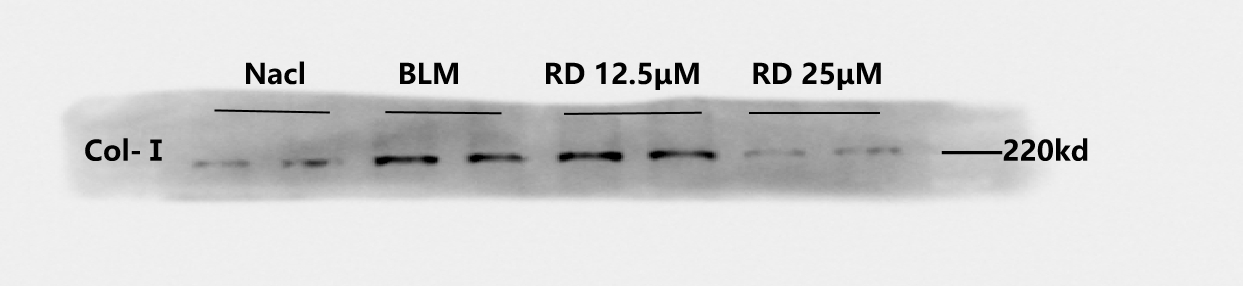
**g**

**BLM**

**+**

**BLM**

**+**


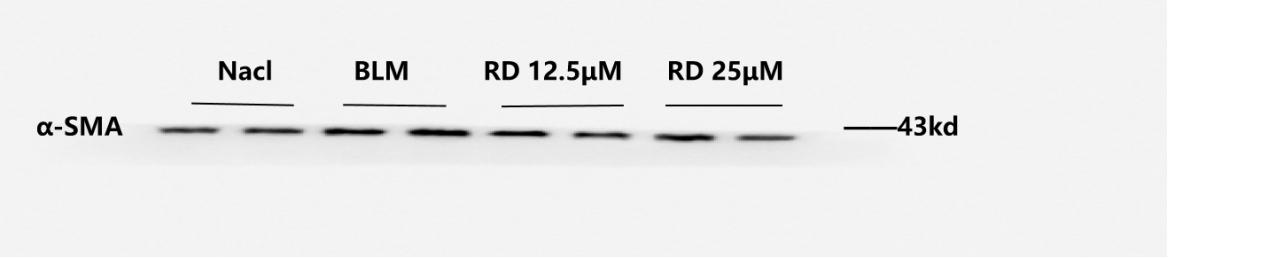


**BLM**

**+**

**BLM**

**+**


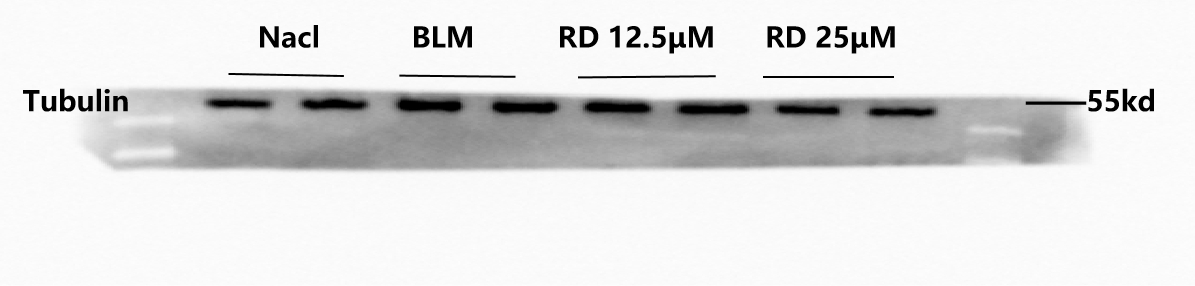


**BLM**

**+**

**BLM**

**+**

**Figure 1.** The original gel of Col-Ⅰ, α-SMA and Tubulin in Figure 1g.


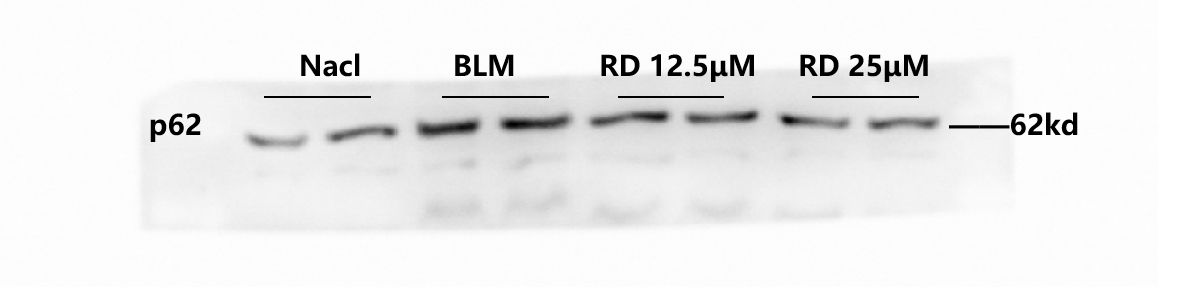
**b**

**BLM**

**+**

**BLM**

**+**

**BLM**

**+**

**BLM**

**+**


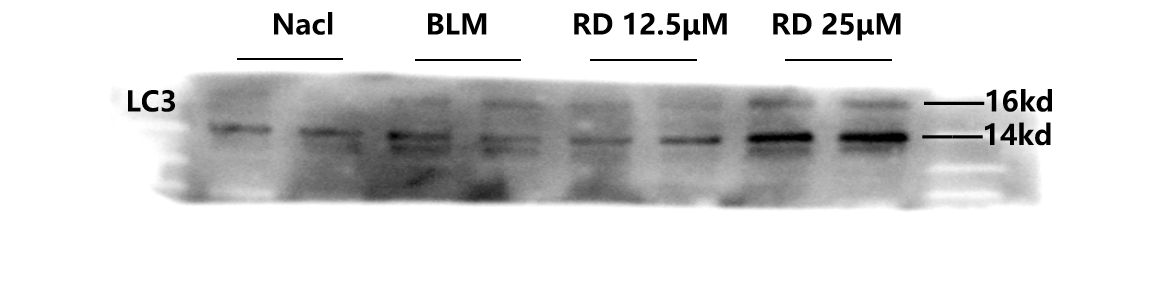


**BLM**

**+**

**BLM**

**+**


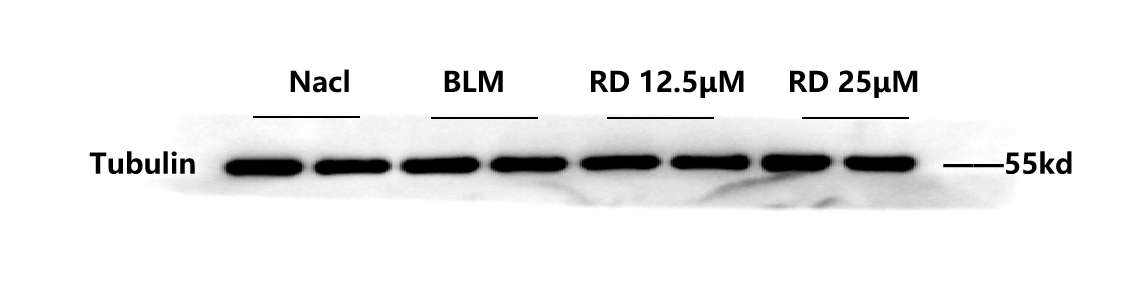


**Figure 3.** The original gel of p62, LC3 and Tubulin in Figure 3b.

**f**


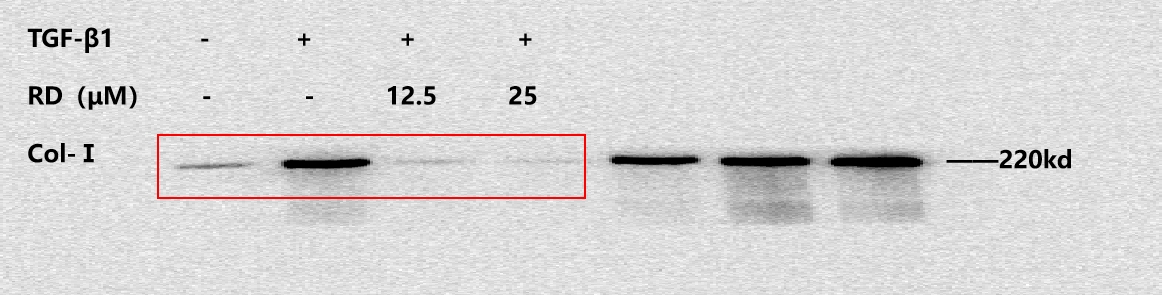


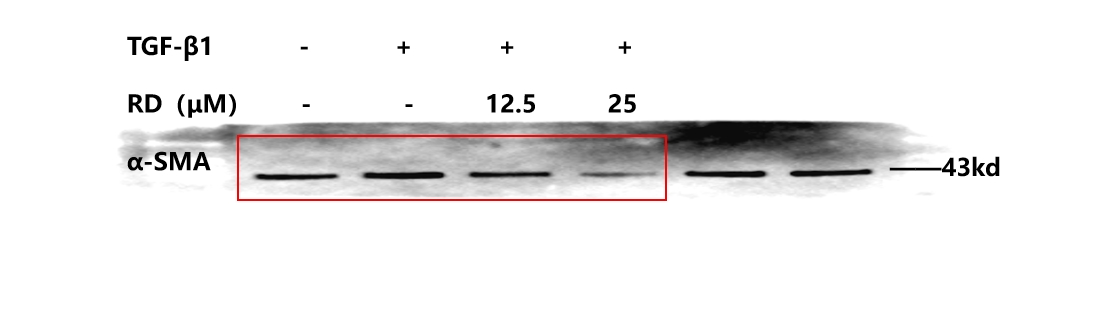


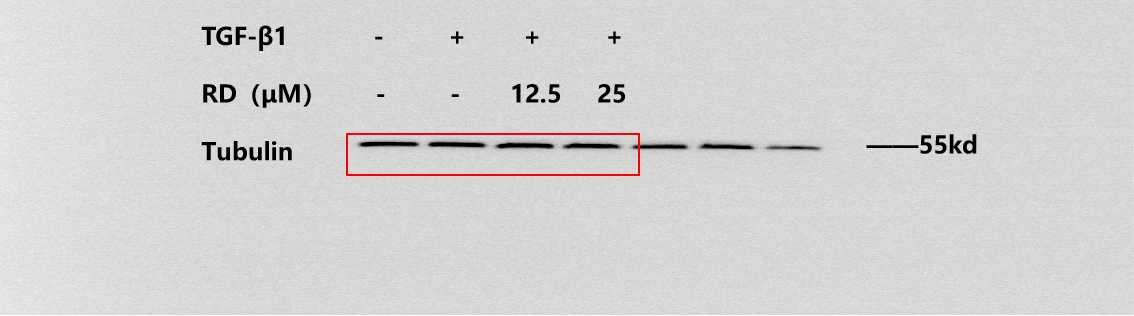


**Figure 4.** The original gel of Col-Ⅰ, α-SMA and Tubulin in Figure 4f.

**f**
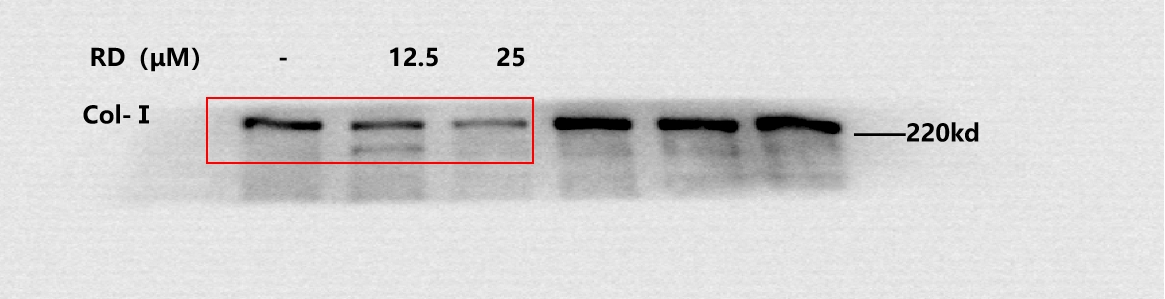


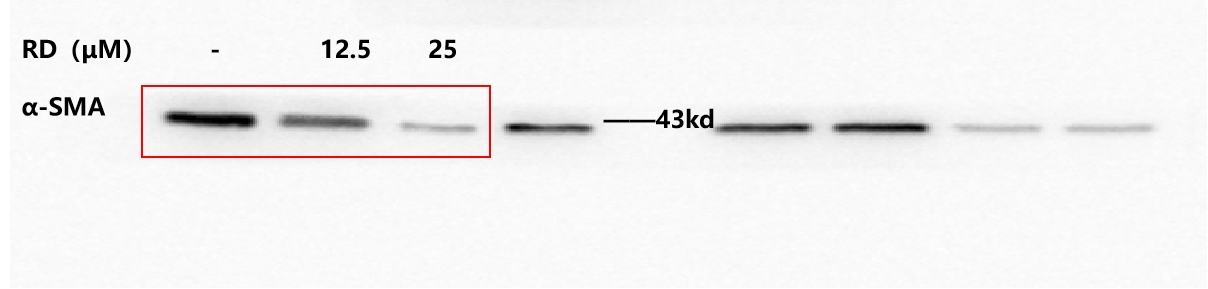


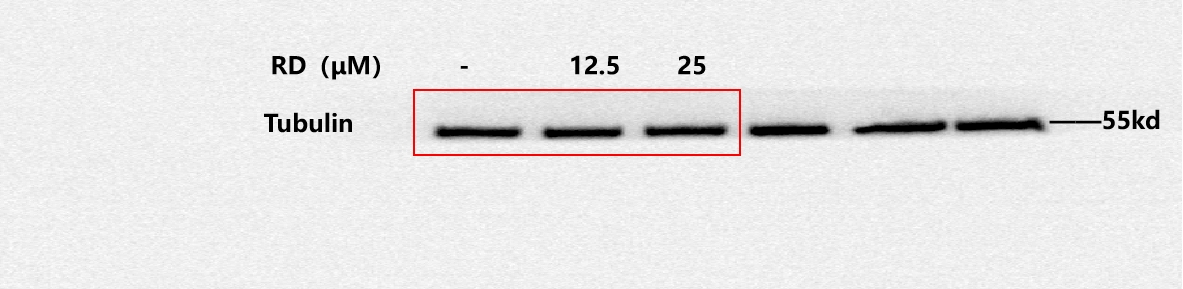


**Figure 5.** The original gel of Col-Ⅰ, α-SMA and Tubulin in Figure 5f.

**a**

**
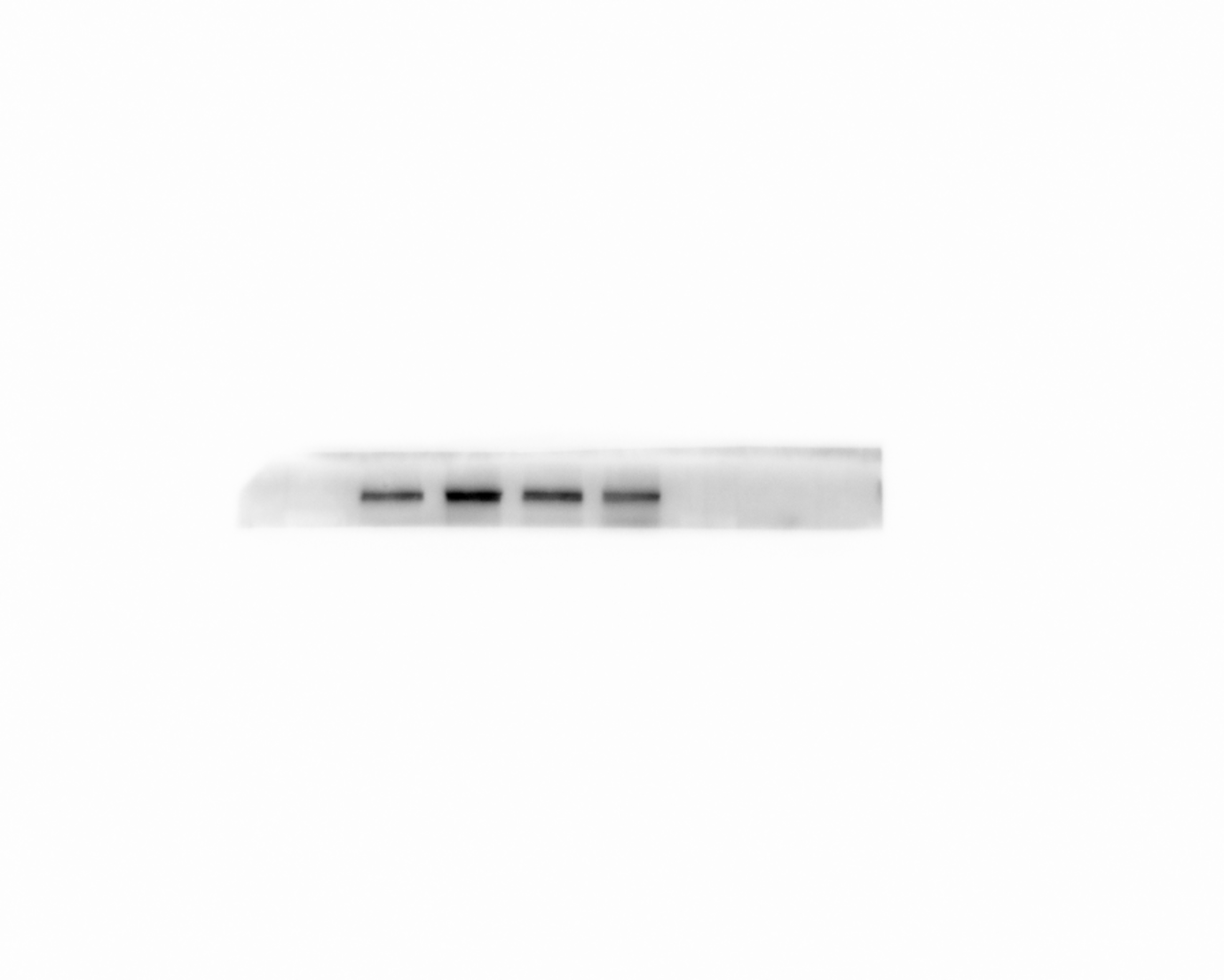
**

**58kd**

**p-Smad3**

**- + + +**

**- - 12.5 25**

**TGF-β1 (5ng/mL)**

**RD (μM)**


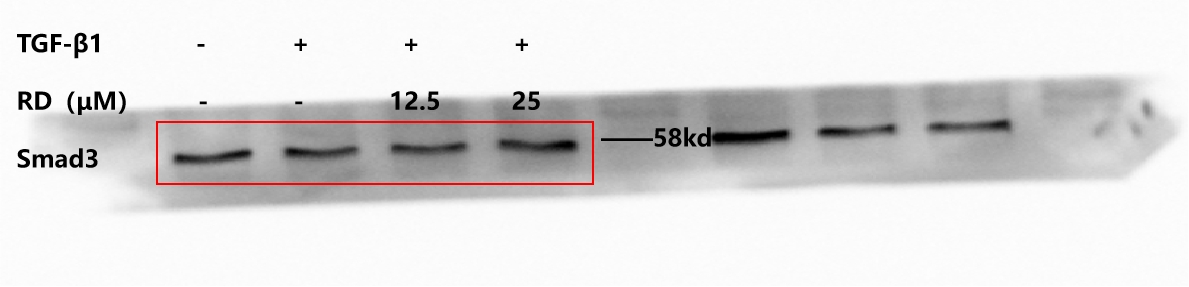


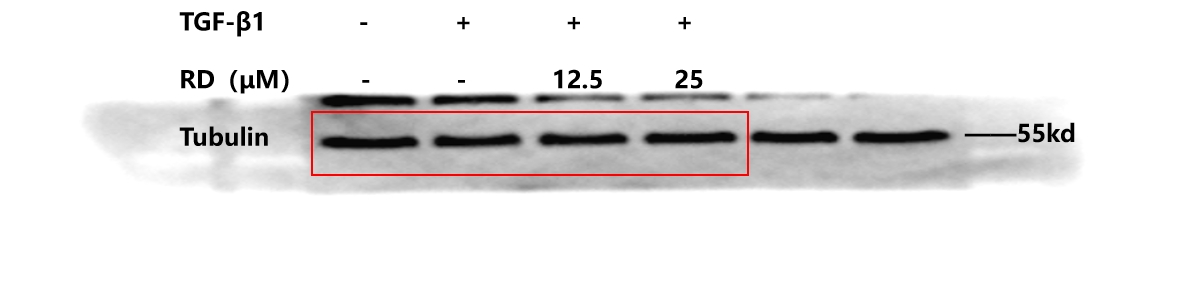


**
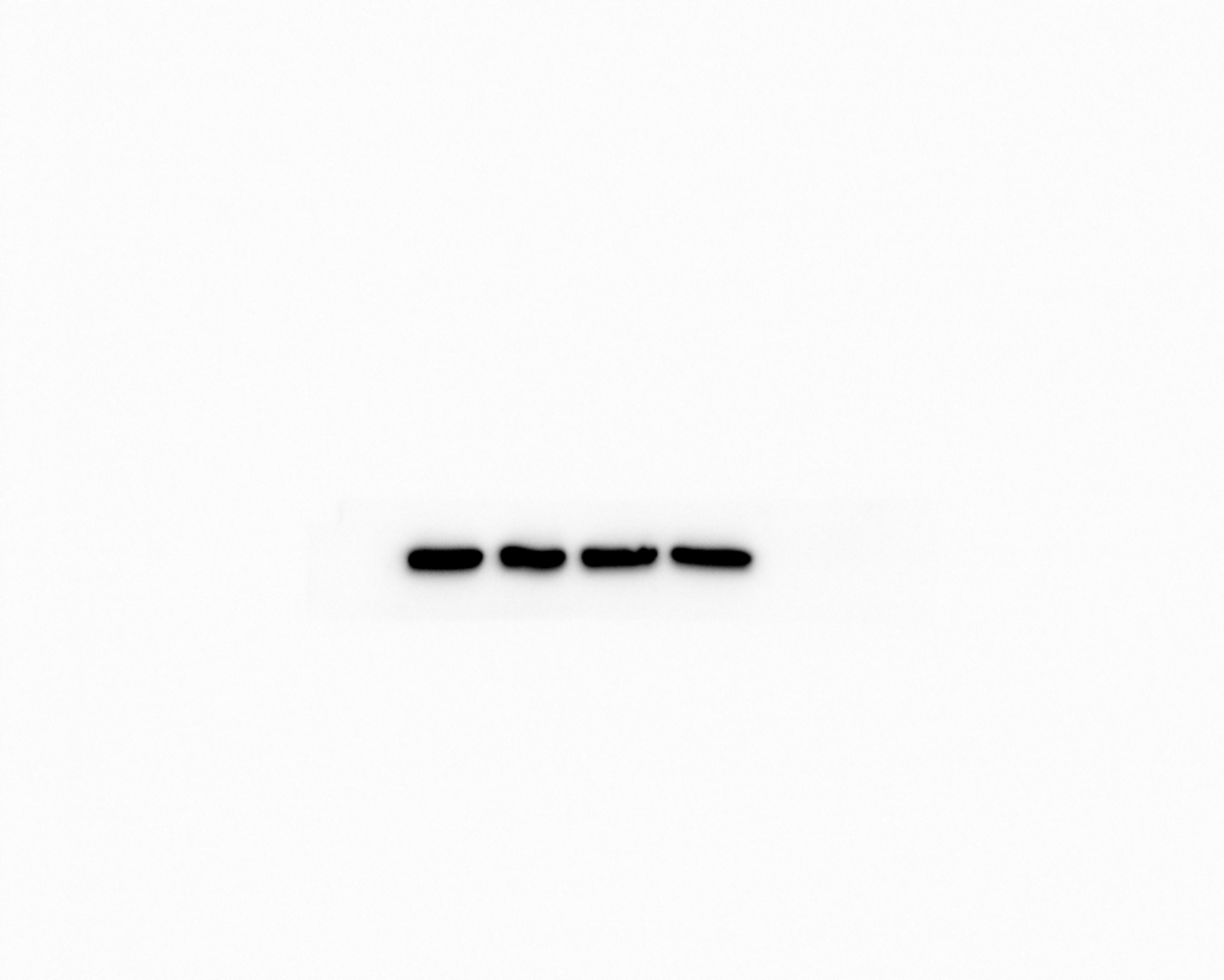
**

**37kd**

**GAPDH**

**- + + +**

**- - 12.5 25**

**TGF-β1 (5ng/mL)**

**RD (μM)**

**Figure 6.** The original gel of p-Smad3 (repeated), Smad3, Tubulin and GAPDH (repeated) in Figure 6a.

**b**
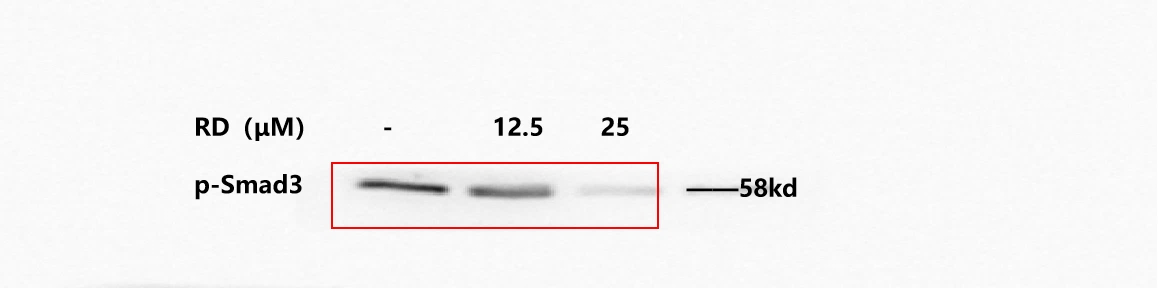


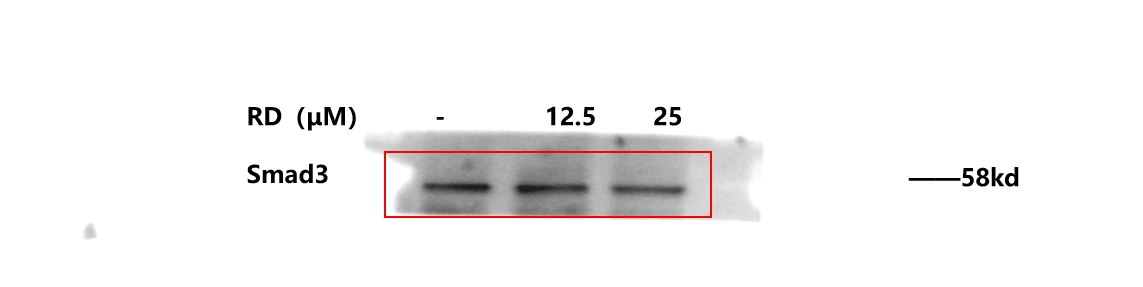


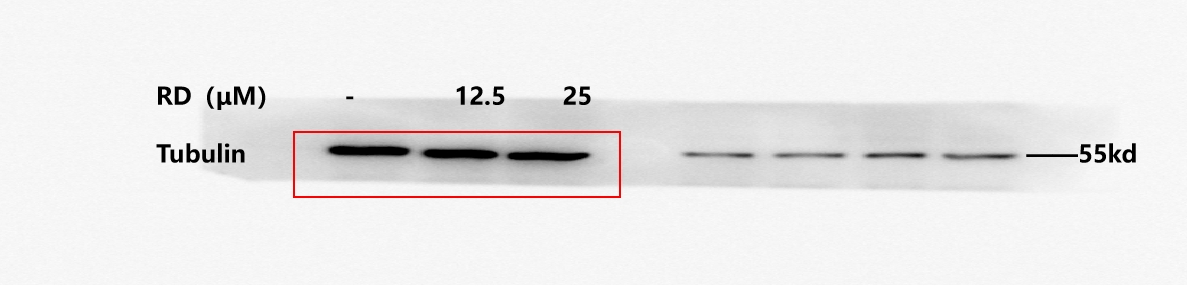


**Figure 6.** The original gel of p-Smad3, Smad3 and Tubulin in Figure 6b.


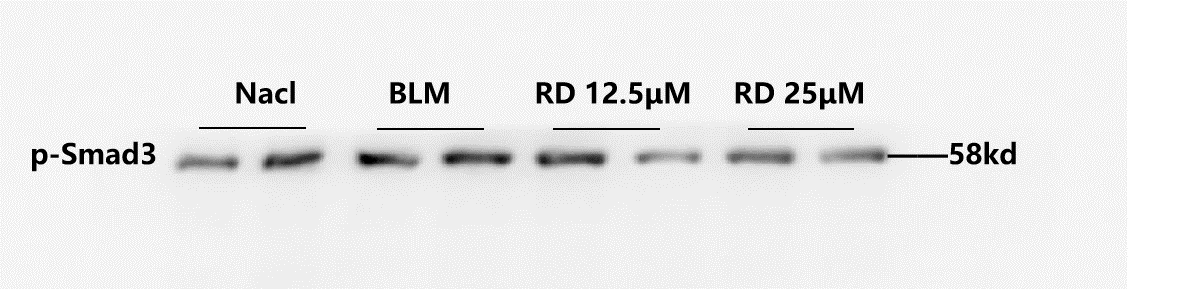
**c**

**BLM**

**+**

**BLM**

**+**

**BLM**

**+**

**BLM**

**+**


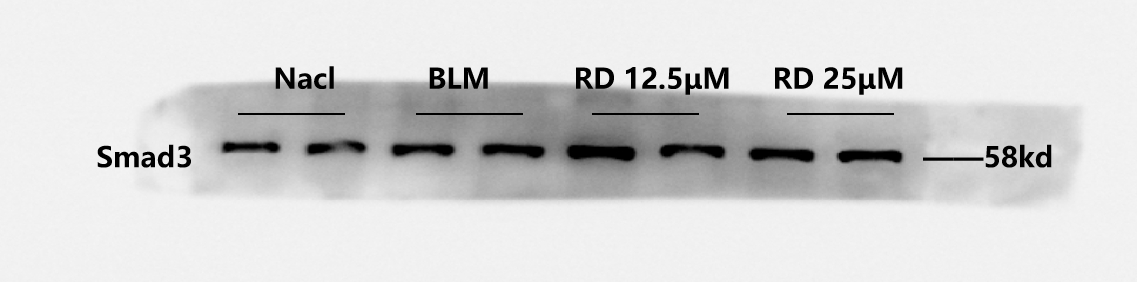


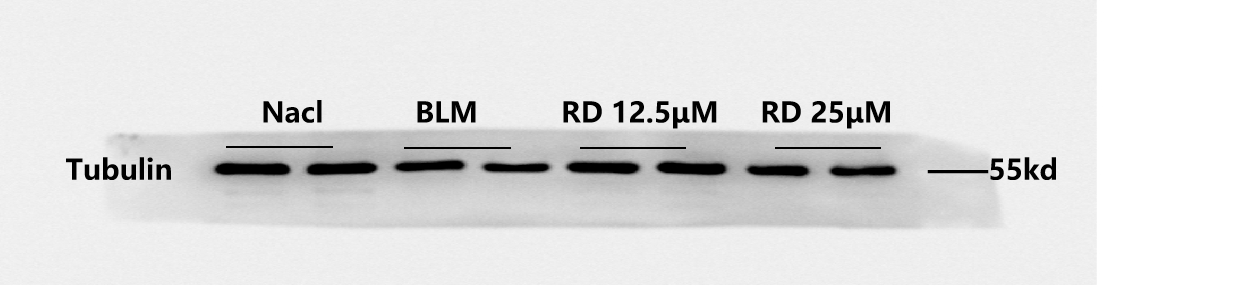


**BLM**

**+**

**BLM**

**+**

**Figure 6.** The original gel of p-Smad3, Smad3 and Tubulin in Figure 6c.

**a**


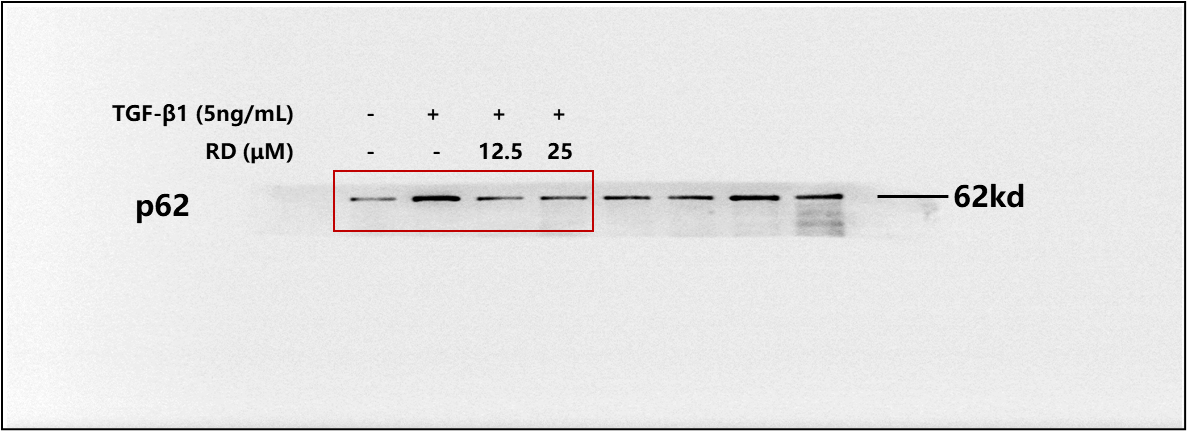


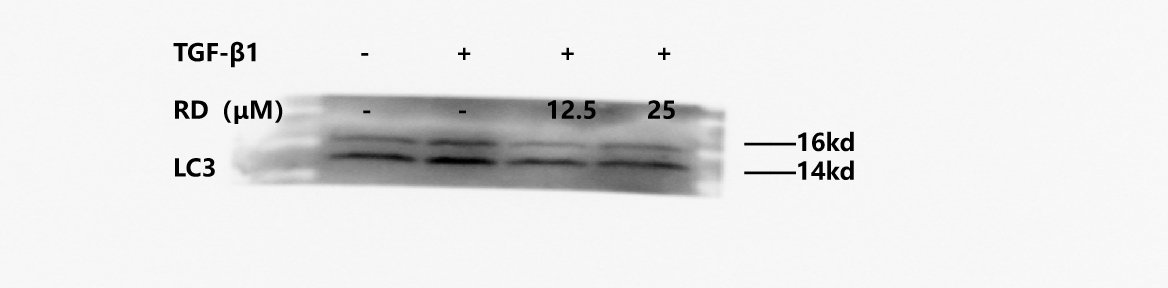


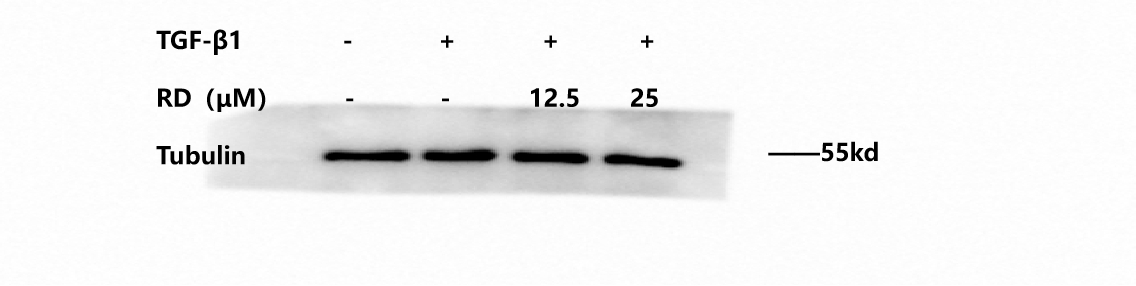


**Figure 7.** The original gel of p62, LC3 and Tubulin in Figure 7a.

**c**
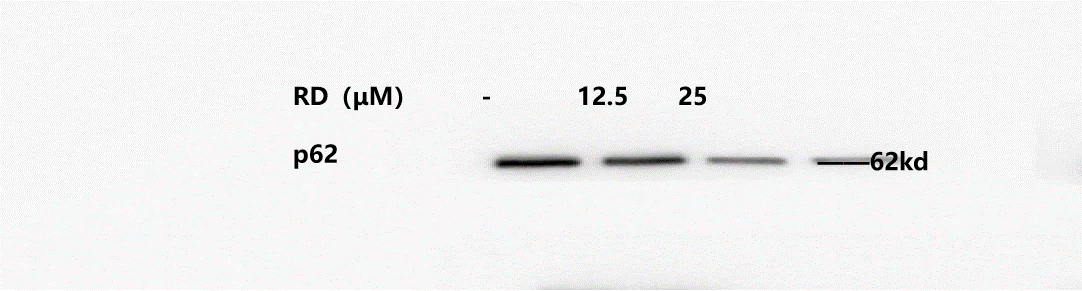


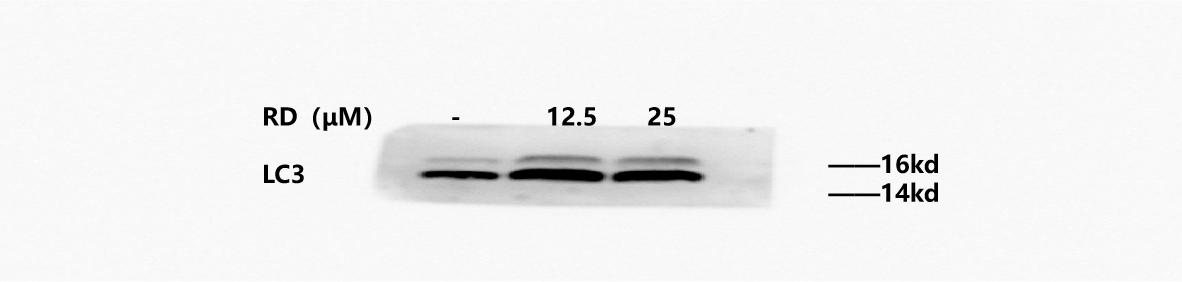


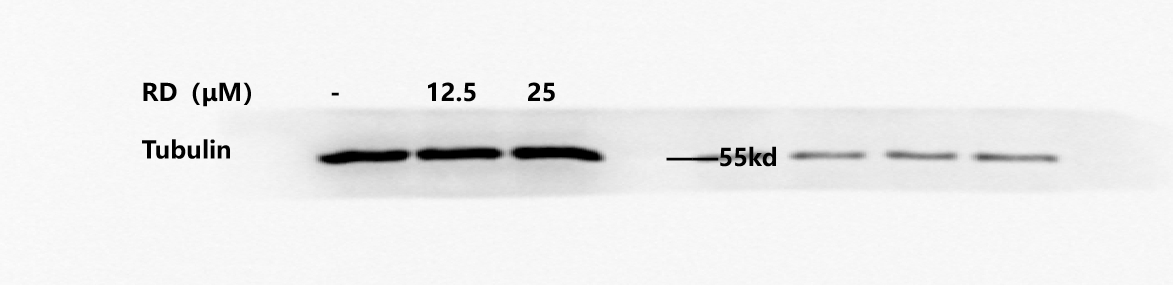


**Figure 7.** The original gel of p62, LC3 and Tubulin in Figure 7c.

**a**
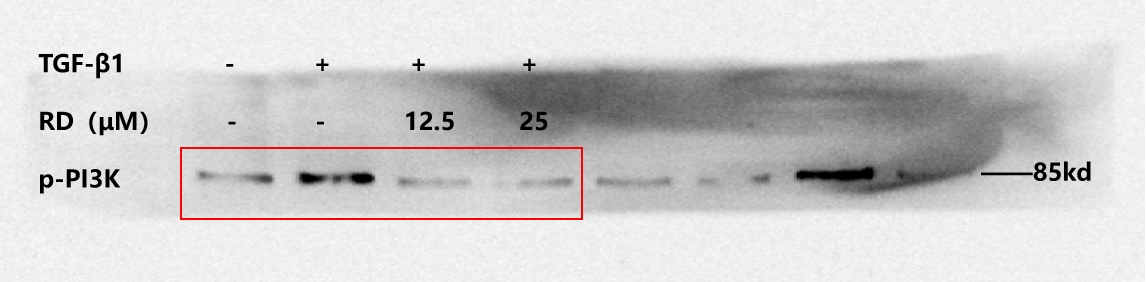

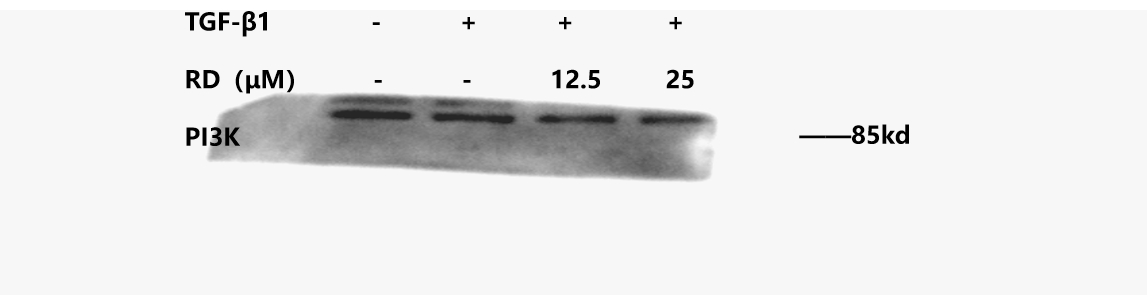

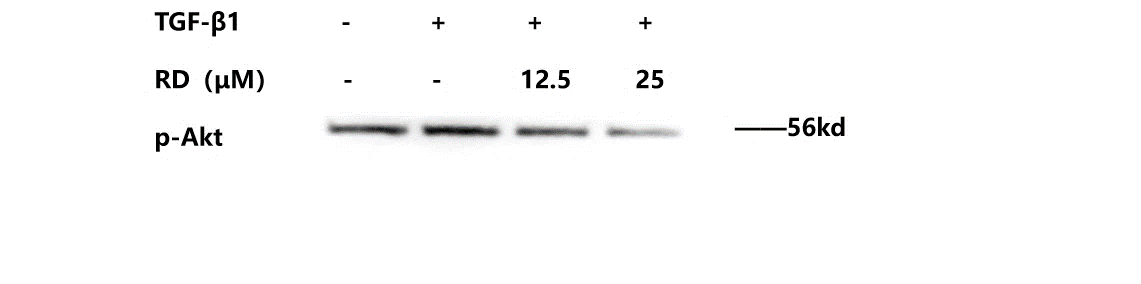


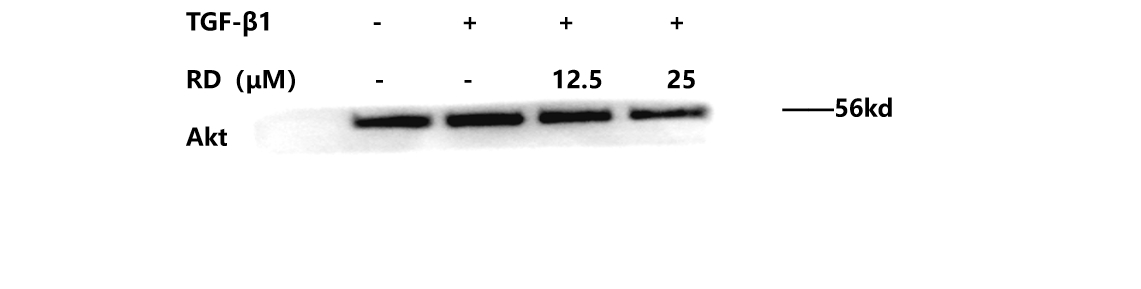


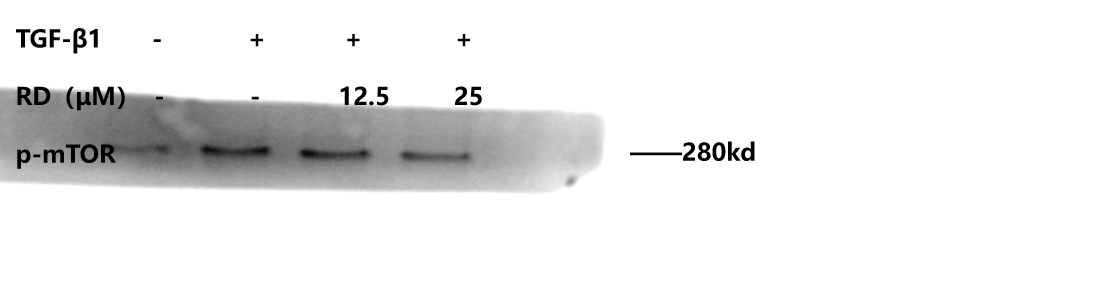

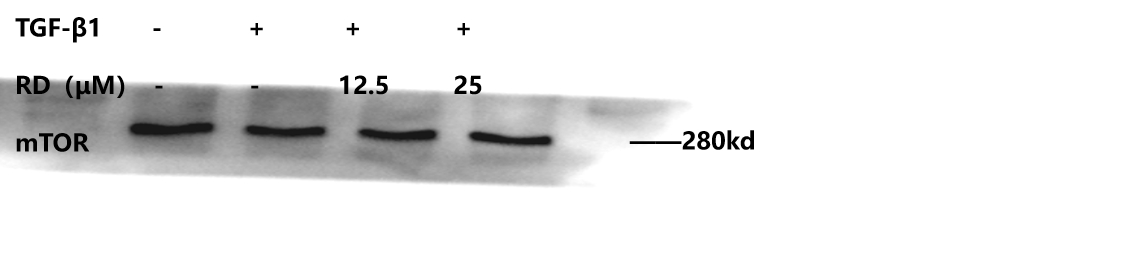

**Figure 8.** The original gel of p-Akt, Akt, p-PI3K, PI3K, p-mTOR, mTOR and Tubulin in Figure 8a.

**b**


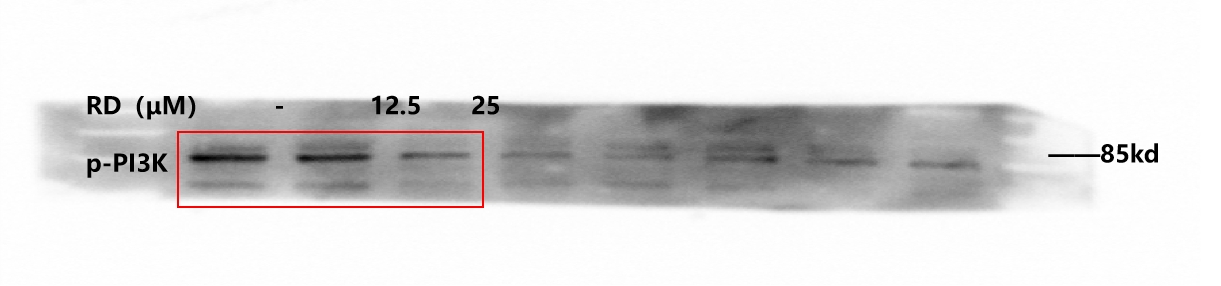


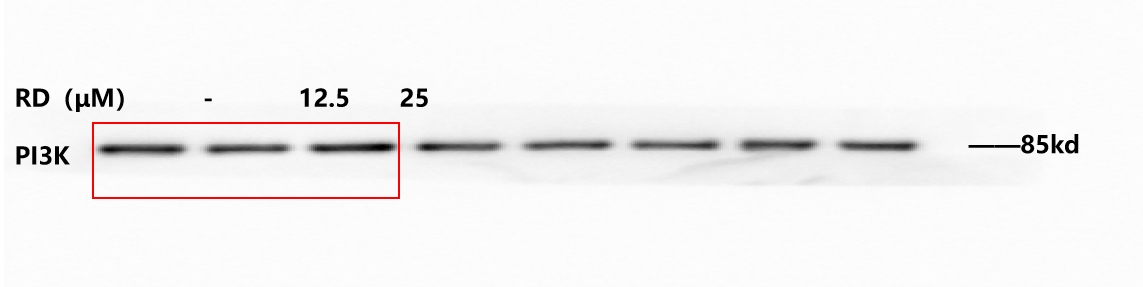


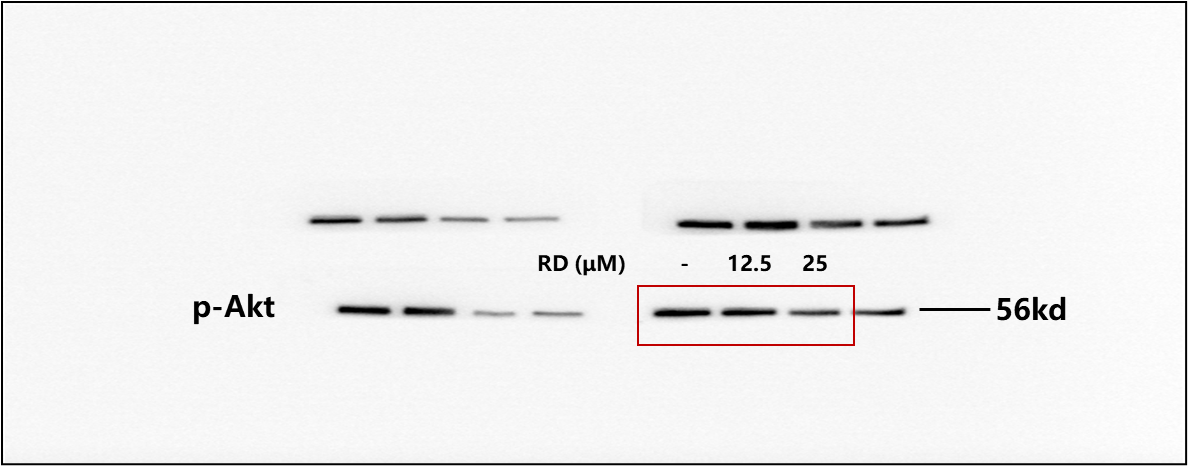


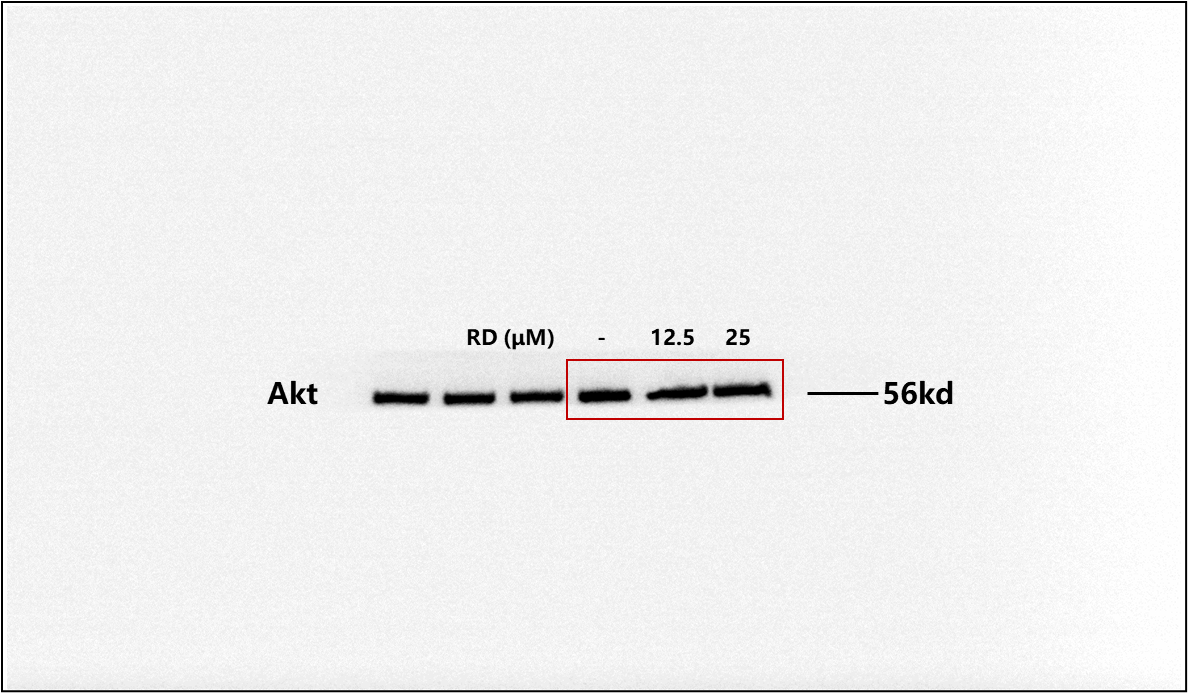


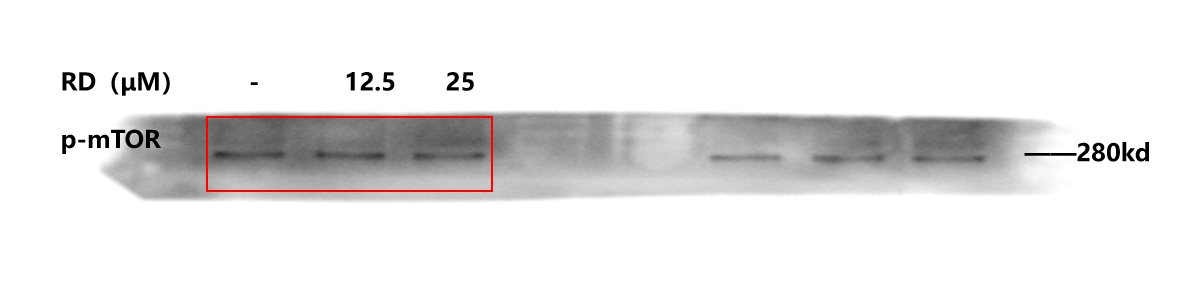


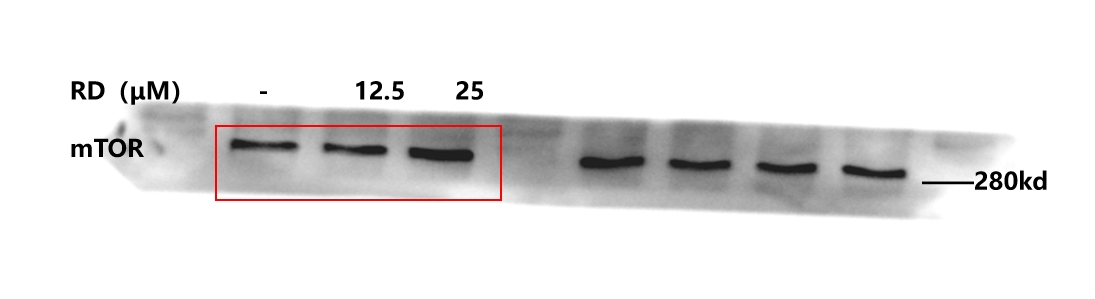


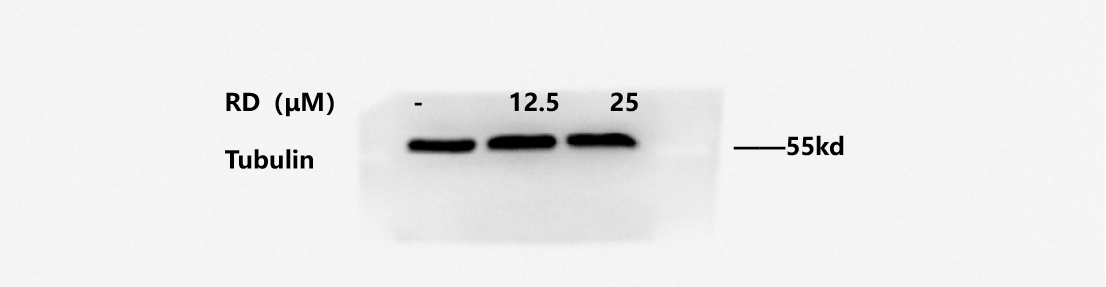


**Figure 8.** The original gel of p-Akt, Akt, p-PI3K, PI3K, p-mTOR, mTOR and Tubulin in Figure 8b.

**
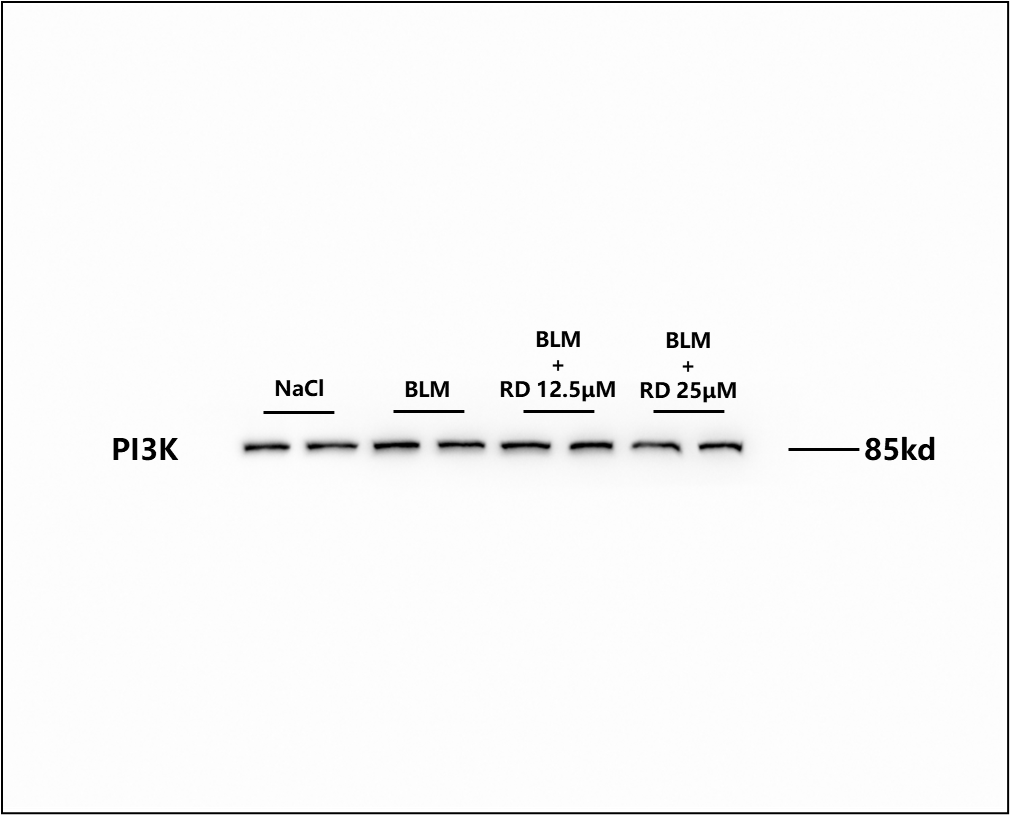
**
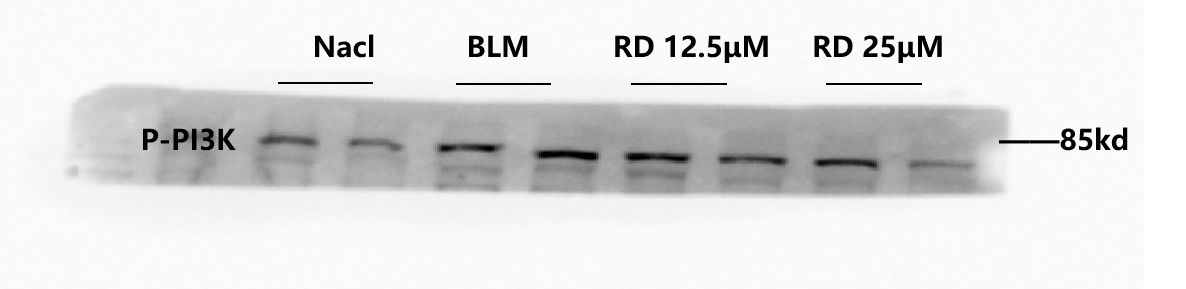
**c**

**BLM**

**+**

**BLM**

**+**


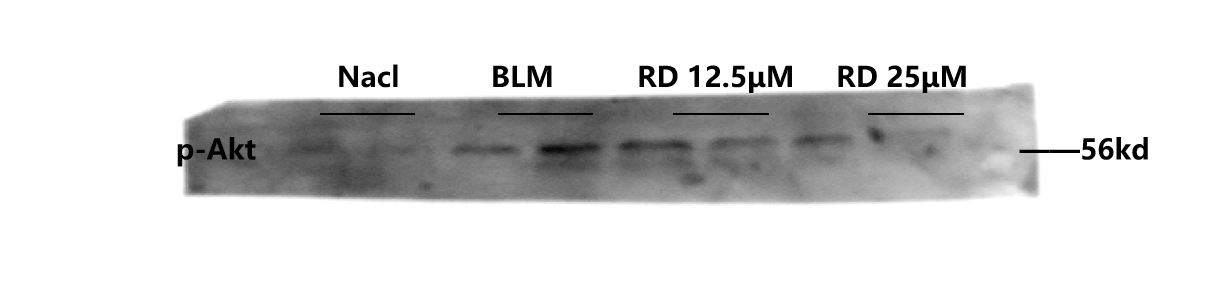


**BLM**

**+**

**BLM**

**+**


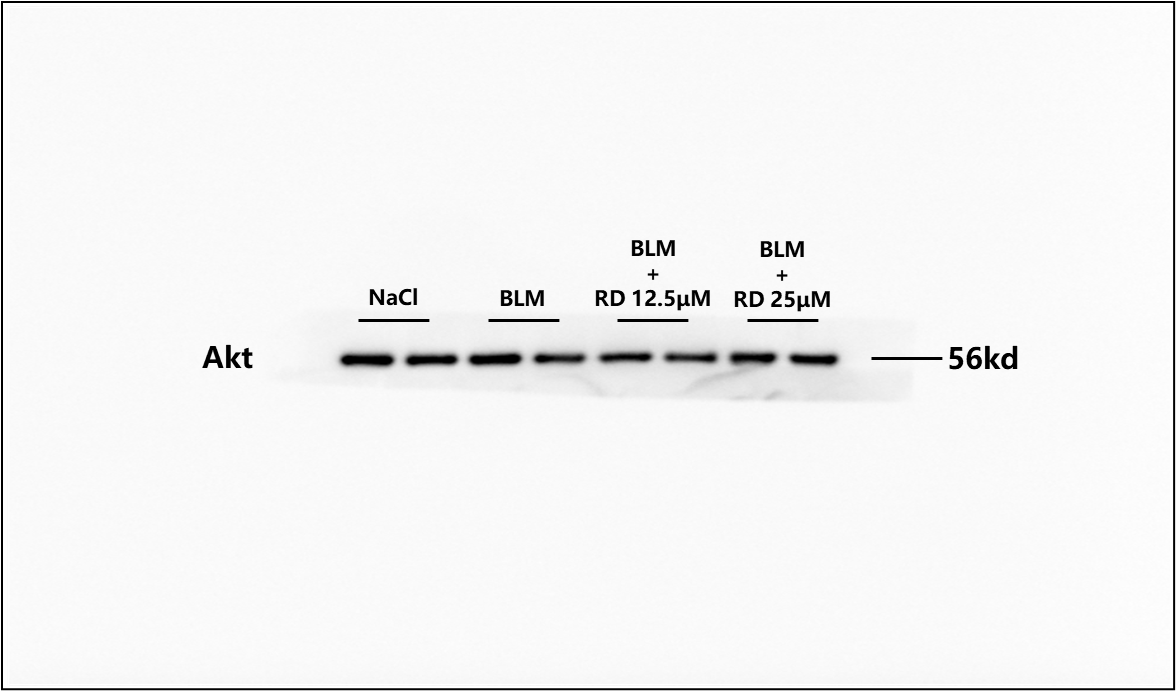


**BLM**

**+**

**BLM**

**+**


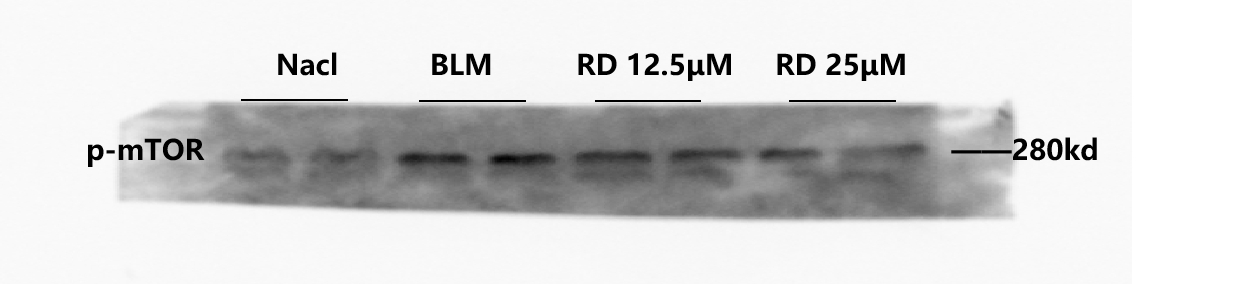


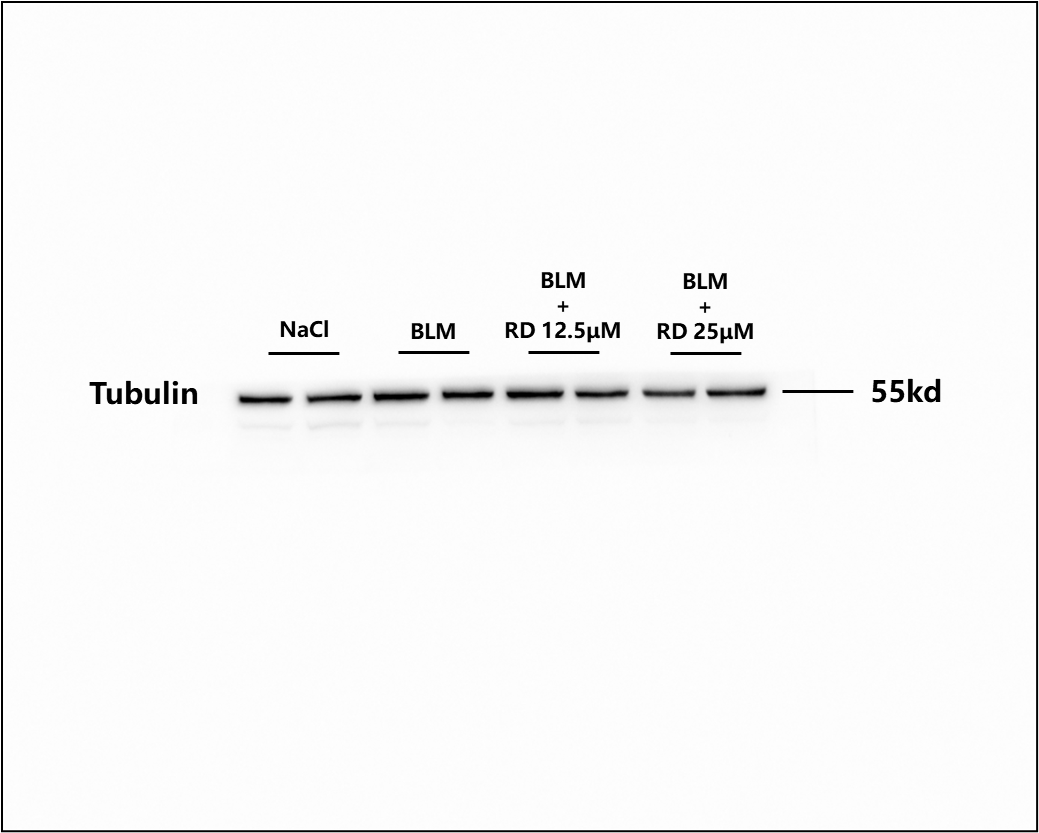

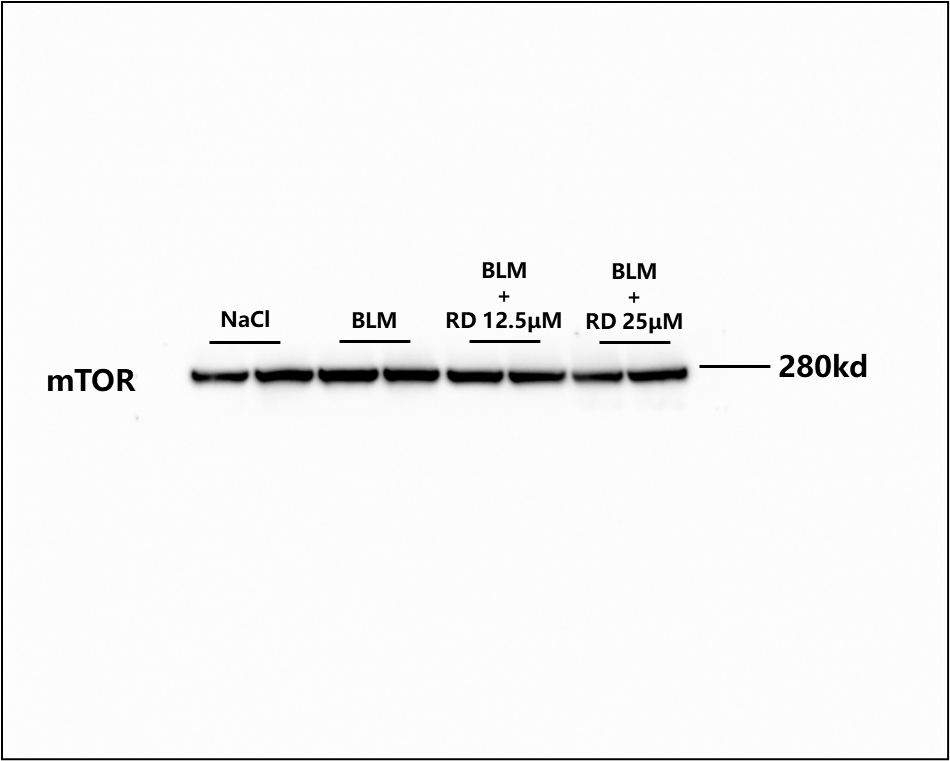


**Figure 8.** The gel of p-Akt, Akt, p-PI3K, PI3K, p-mTOR, mTOR and Tubulin in Figure 8c.
